# Exploring Three PIPs and Three TIPs of Grapevine for Transport of Water and Atypical Substrates through Heterologous Expression in *aqy-null* Yeast

**DOI:** 10.1371/journal.pone.0102087

**Published:** 2014-08-11

**Authors:** Farzana Sabir, Maria José Leandro, Ana Paula Martins, Maria C. Loureiro-Dias, Teresa F. Moura, Graça Soveral, Catarina Prista

**Affiliations:** 1 Centre for Botany Applied to Agriculture (CBAA), Instituto Superior de Agronomia, University of Lisbon, Lisbon, Portugal; 2 Instituto de Investigação do Medicamento (iMed.ULisboa), Faculdade de Farmácia, Universidade de Lisboa, Lisboa, Portugal; 3 Dept. de Bioquímica e Biologia Humana, Faculdade de Farmácia, Universidade de Lisboa, Lisboa, Portugal; Technion-Israel Institute of Technology Haifa 32000 Israel, Israel

## Abstract

Aquaporins are membrane channels that facilitate the transport of water and other small molecules across the cellular membranes. We examined the role of six aquaporins of *Vitis vinifera* (cv. Touriga nacional) in the transport of water and atypical substrates (other than water) in an *aqy-null* strain of *Saccharomyces cerevisiae*. Their functional characterization for water transport was performed by stopped-flow fluorescence spectroscopy. The evaluation of permeability coefficients (*P_f_*) and activation energies (*Ea*) revealed that three aquaporins (*Vv*TnPIP2;1, *Vv*TnTIP1;1 and *Vv*TnTIP2;2) are functional for water transport, while the other three (*Vv*TnPIP1;4, *Vv*TnPIP2;3 and *Vv*TnTIP4;1) are non-functional. TIPs (*Vv*TnTIP1;1 and *Vv*TnTIP2;2) exhibited higher water permeability than *Vv*TnPIP2;1. All functional aquaporins were found to be sensitive to HgCl_2_, since their water conductivity was reduced (24–38%) by the addition of 0.5 mM HgCl_2_. Expression of *Vitis* aquaporins caused different sensitive phenotypes to yeast strains when grown under hyperosmotic stress generated by KCl or sorbitol. Our results also indicate that *Vitis* aquaporins are putative transporters of other small molecules of physiological importance. Their sequence analyses revealed the presence of signature sequences for transport of ammonia, boron, CO_2_, H_2_O_2_ and urea. The phenotypic growth variations of yeast cells showed that heterologous expression of *Vitis* aquaporins increased susceptibility to externally applied boron and H_2_O_2_, suggesting the contribution of *Vitis* aquaporins in the transport of these species.

## Introduction

Aquaporins play a crucial role in maintaining water and ion homeostasis of plants, essential for plant cell integrity, growth and survival in their ever-changing environment. These water channels can provide rapid and reversible changes to cells hydraulic conductance by modulating membrane water permeability [1]. Aquaporins belong to Major Intrinsic Proteins (MIPs) family and based on their sequence similarity and sub-cellular localization, plant aquaporins are divided in seven subfamilies: the plasma membrane intrinsic proteins (PIPs), the tonoplast intrinsic proteins (TIPs), the nodulin-26-like intrinsic proteins (NIPs), the small intrinsic proteins (SIPs), the GlpF-like intrinsic proteins (GIPs), the hybrid intrinsic proteins (HIPs) and the uncategorized X intrinsic proteins (XIPs) [2]. Studies on plant aquaporins revealed their role far beyond the membrane water transport. Besides water, they are reported to transport also other small molecules and/or gases of physiological importance (reviewed by [3]), suggesting their versatile functions in plants. Putative substrate specificities of aquaporins are generally assigned by the presence of specific amino acid residues at well-defined positions in the sequences [4].

Since aquaporins establish a tight association between water transport and plant development and adaptation under stress conditions, a rigorous regulation of aquaporin activity is essential to fine-tune the overall hydraulic conductivity in plants [5]. Expression of aquaporin genes can be altered under various environmental conditions as well as according to cell/tissue type and plant developmental stages [6]. Besides these initial regulatory steps of gene expression, the activity of translated and targeted aquaporin proteins can be further regulated by various post-translational modifications such as methylation, glycosylation, phosphorylation, membrane trafficking, heteromerization, and their gating can also be regulated by pH, divalent ions and membrane tension [7]. Various stress conditions like anoxia, salt and water stress have also been reported to affect the activity of aquaporins in plants (reviewed by [3], [6]).

*Vitis vinifera* cv. Touriga nacional is an important Portuguese cultivar. This variety is a key ingredient in both dry red and fortified wines (particularly, Port wine). Grapevines are known to be extremely stress-tolerant plants, especially for dry environment [8]. In fact, deficit irrigation techniques are commonly used to achieve high fruit quality [9]. Since the water status of the plant greatly influences the fruit quality and hence the characteristics of wine [10], it is significant to study the molecular cell entry point of water, i.e. aquaporins in these plants. Release of full genomic sequence of grapevine revealed the occurrence of 28 genes encoding putative aquaporins in *V. vinifera* [11]. Comprehensive phylogenetic analyses of the deduced amino acid sequences suggest that the *V. vinifera* (cv. Cabernet Sauvignon and cv. Pinot Noir) aquaporins can be distributed in the four main subfamilies: PIPs (8 genes), TIPs (10 genes), NIPs (8 genes) and SIPs (2 genes) [12]. Despite being a very important economical plant, only few reports are available on *Vitis* aquaporins, explaining their quantitative expression in various rootstocks [13], during water stress [14], [15] and their cloning and expression *in planta* [8], [16] or in heterologous systems, such as *Xenopus* oocytes [1], [12] and *S. cerevisiae* [17], [18]. Although from a molecular point of view the presence of aquaporins can be easily recognized in a genome, their physiological role *in planta* is still difficult to understand. At transcript level, plant aquaporins respond variedly to stress, depending on the plant tissue/organ, cultivars/species and types/degree of stress [1], impairing the interpretation of the role of an individual aquaporin. To study the physiological role of each aquaporin, we used *S. cerevisiae* as a simple and well-characterized heterologous expression system [19]. The evaluation of water transport activity in intact yeast cells through stopped-flow fluorescence spectroscopy is already well-established [17], [20].

The present work is focused on the cloning and expression of putative aquaporins (three PIPs and three TIPs) of *Vitis vinifera* (cv. Touriga nacional) in an *aqy-null* strain of *S. cerevisiae*. Their functional characterization for water transport was performed through stopped-flow spectroscopy. Further, tolerance/sensitivity of these aquaporins expressing strains was tested under hyperosmotic stress exerted by KCl or sorbitol. This study also includes the analysis of signature sequences for transport of atypical substrates and growth assays of yeast strains expressing *Vitis* aquaporins in the presence of these substrates, to explore their putative route of transport.

## Materials and Methods

### Yeast strain, plasmid and growth conditions

*Saccharomyces cerevisiae* 10560-6B *MATα leu2::hisG trp1::hisG his3::hisG ura3-52 aqy1::KanMX4 aqy2::HIS3* (from now on designated as *aqy-null*) was used as host strain for heterologous expression of putative aquaporins from *V. vinifera* cv. Touriga nacional. The centromeric plasmid pUG35 was used for cloning, conferring C-terminal GFP tagging, MET25 promoter and CYC1-T terminator [21]. For propagation of these plasmids, *Escherichia coli* DH5α strain was used as host [22]. *E. coli* transformants were grown in Luria-Bertani (LB) medium supplemented with ampicillin (100 µg ml^−1^), at 37°C. The host *S. cerevisiae* strain (*aqy-null*) was maintained in YPD medium (5 g l^−1^ yeast extract, 10 g l^−1^ peptone, 20 g l^−1^ glucose and 20 g l^−1^ agar). Transformed yeast strains were grown and maintained in YNB medium without amino acids (DIFCO) with 2% (w/v) glucose supplemented with the adequate requirements for prototrophic growth [23].

### Cloning and heterologous expression of *Vitis vinifera* aquaporins in *S. cerevisiae*

To clone the putative aquaporins from *V. vinifera* cv. Touriga nacional, their cDNA (kindly provided by Dr. Luísa Carvalho, ISA-ULisboa) amplification, cloning and expression in *S. cerevisiae* were performed according to previously described methods [17]. Primers used in this study are listed in Table S1.

### Sequence analysis

Nucleotide sequences identified in the present study are submitted to National Center for Biotechnological Information (NCBI) (http://www.ncbi.nlm.nih.gov). These sequences were translated by ExPASy translate tool (http://web.expasy.org/translate/). Deduced amino acid sequences were analysed and compared with database sequences of *V. Vinifera* cv. Pinot noir, available at Grape Genome Browser (http://www.genoscope.cns.fr/externe/Genome Browser/Vitis/) and NCBI. Multiple protein sequence alignments were generated by using the ClustalX [24] and BioEdit [25] programs. Phylogenetic tree was constructed from the alignment of deduced amino acid sequences obtained from the present study and our previous study [17] with twenty-eight amino acid sequences of aquaporins of *V. vinifera* cv. Pinot noir, by MEGA5.1 software using neighbor-joining method [26]. Topology and hydrophobicity of deduced amino acid sequences were predicted by using various ExPASy tools e.g. TMHMM [27], HMMTOP [28] and TMPred [29].

All gene sequences are available from at GeneBank VvTnPIP1;4 accession = KJ697714, VvTnPIP2;1 accession = KJ697715, VvTnPIP2;3 accession = KJ697716, VvTnTIP1;1 accession = KJ697717, VvTnTIP2;2 accession = KJ697718, VvTnTIP4;1 accession = KJ697719.

To search the signature sequences for transport of atypical substrates, obtained amino acid sequences were grouped with reference sequences as per their reported substrates for transport, described by [4] and were aligned using ClustalX. Conserved amino acid residues at NPA, ar/R constriction and P1–P5 regions were determined from the alignment and matched with reference sequences of transporters of various substrates. Based on the conserved residues at these positions, selectivity profiles of cloned aquaporins were predicted.

### Water transport assays of *Vitis* aquaporins expressed in *S. cerevisiae*

Functional analyses of heterologously expressed *Vitis* aquaporins for water conductivity were performed by stopped-flow fluorescence spectroscopy as previously described [17]. Briefly, yeast strains grown in liquid YNB medium were harvested at mid exponential phase (OD_640 nm_≈1.0) (Ultrospec 2100 pro, Amersham Biosciences) and incubated for 1 hour at 28°C in YPD medium (6 g l^−1^ wet weight). Further, cells were washed and re-suspended in ice-cold 1.4 M sorbitol (3 ml g^−1^ wet weight) and incubated on ice for at least 90 minutes. Cells were pre-loaded with the membrane permeable nonfluorescent precursor 5-(and-6)-carboxyfluorescein diacetate (CFDA), which is intracellularly hydrolyzed releasing the membrane impermeable fluorescent form. For water transport assays, hyperosmotic shocks were applied on stopped-flow apparatus (HI-TECH Scientific PQ/SF-53). Cells equilibrated with 1.4 M sorbitol were mixed with an equal volume of 2.1 M sorbitol. The resulting cell shrinkage caused a quenching of the fluorescence intensity. Signals were fitted to a single exponential, from which the rate constant (*k*) was calculated. Permeability coefficient (*P_f_*) and activation energy (*Ea*) were estimated as described by [17], [20].

Inhibition of water transport in *Vitis* aquaporins expressing yeast strains was tested with mercury chloride (HgCl_2_), a well-known inhibitor of aquaporins. Yeast strains expressing functional *Vitis* aquaporins were incubated with HgCl_2_ (0.5 and 1 mM, in 50 mM potassium citrate buffer, pH 5.0) for 15 and 30 minutes before the osmotic shock at 23°C. The yeast strain with the empty plasmid was treated in the same way and considered as control. Osmotic shocks were applied without and after incubation with HgCl_2_ and the signals obtained were compared and used to calculate the *P_f_* in both conditions.

### Growth assays under osmotic stress and sensitivity tests on atypical substrates

*S. cerevisiae* strains harboring *Vitis* aquaporins were tested for their ability to grow under osmotic stress. Moreover, possibility to transport atypical substrates, such as ammonia, boron, hydrogen peroxide (H_2_O_2_) and urea was also investigated. Growth assays were performed on solid YNB medium (pH 5.0) supplemented with 2% (w/v) glucose. NaCl (0.5, 1.0 and 1.5 M), KCl (0.5, 1.0 and 1.5 M) and osmoequivalent concentration of sorbitol (0.84, 1.4 and 2.1 M) were added to growth media for osmotic stress experiments. Hydrogen peroxide (0.5, 0.75, 1.0, 1.5 and 2.0 mM) and boron (as boric acid, 20, 40, 50 and 60 mM), were used as atypical substrates for transport. Media with H_2_O_2_ was freshly prepared at the time of inoculation. Moreover, in order to test the putative transport of ammonia (1.0, 2.0 and 3.0 mM) and urea (1.0, 2.0, 2.5 and 3.5 mM), either of them was used as sole nitrogen source. Yeast strains were initially grown in liquid YNB medium supplemented with 2% (w/v) glucose, with orbital shaking (180 rpm) at 28°C, up to OD_640 nm_≈1.0 corresponding to 1×10^7^ cells/ml. Cells were centrifuged and washed in sterile distilled water and re-suspended to OD_640 nm_≈10. Multi-well plates were prepared with serial 10-fold dilutions of the original concentrated culture, 3 µl suspensions was spotted with replica platter for 96-well plates device on plates containing YNB solid medium with ammonia, boric acid, H_2_O_2_ and urea, separately and incubated at 28°C. Strain with the empty plasmid (pUG35) was considered as control. Differences in growth phenotypes of yeast strains were recorded after 1 and 2 weeks of incubation.

### Microscopy

For the localization of GFP-tagged proteins in *S. cerevisiae*, mid-exponential phase cells were observed under Leitz Wetzlar Germany 513558 epifluorescence microscope equipped with a Leitz Wetzlar Germany Type 307-148002 514687 mercury bulb and BP 340–380; BP 450–490 (for GFP visualizing); BP 515–560 filter sets. Images were obtained with a digital camera Axiocam Zeiss using AxioVision Rel. 4.8.2 Software.

### Statistical analysis

All the data were collected from at least three independent experiments. For stopped-flow experiments, usually five runs at each temperature and ten runs for *P_f_* at 23°C were stored and analyzed in each experiment. Student's t test was used for statistical analysis. P<0.05 (marked as *) was considered to be statistically significant. Data are presented as mean ± standard deviation (SD).

## Results

### Cloning and heterologous expression of *Vitis vinifera* aquaporins in *S. cerevisiae*

**Figure 1:**
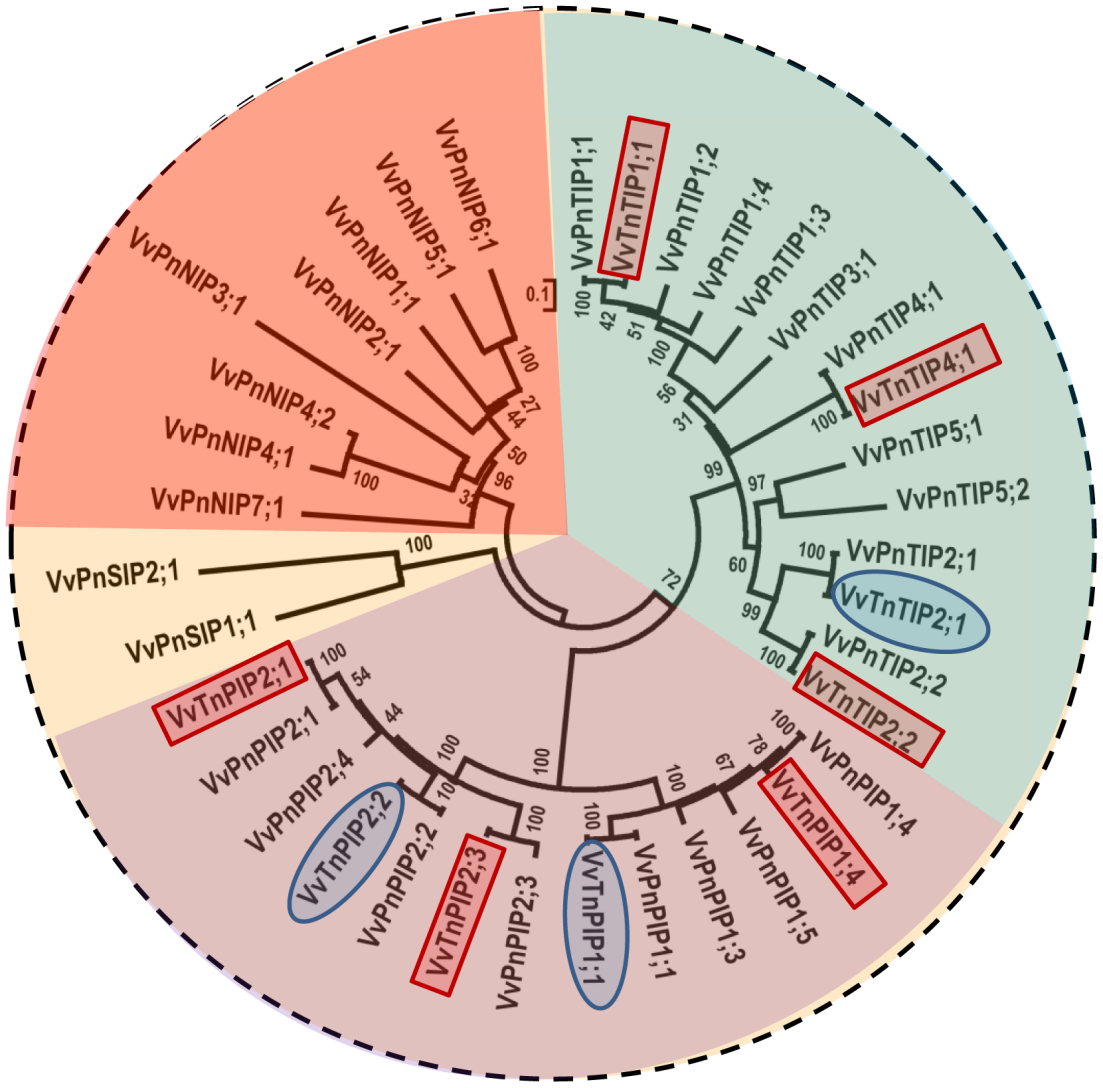
Phylogenetic tree based on protein sequences of aquaporins from *V. vinifera* cv. Pinot noir and cv. Touriga nacional. Dendrogram depicting the phylogenetic relationship between the aquaporins of Touriga nacional variety cloned in this study (framed in rectangular shape), previous study (oval shape frame) [17] with the aquaporins of Pinot noir variety. Dendogram was generated by neighbor-joining method (applied to 1000 bootstrap data sets) using the MEGA5.1 program [26]. Accession numbers of presented protein sequences are: *Vv*PnPIP1;1 (CAO41326, GSVIVT00029248001), *Vv*PnPIP1;3 (CAO62835, GSVIVT00000433001), *Vv*PnPIP1;4 (CAO39626, GSVIVT00026881001), *Vv*PnPIP1;5 (CAO39627, GSVIVT00026882001), *Vv*PnPIP2;1 (CAN75442), *Vv*PnPIP2;2 (CAO47394, GSVIVT00036133001), *Vv*PnPIP2;3 (CAO18152, GSVIVT00023192001), *Vv*PnPIP2;4 (CAO21844, GSVIVT00024536001), *Vv*PnTIP1;1 (CAO69259, GSVIVT00018548001), *Vv*PnTIP1;2 (CAO63006, GSVIVT00000605001), *Vv*PnTIP1;3 (CAO16745, GSVIVT00022146001), *Vv*PnTIP1;4 (CAO21720, GSVIVT00024394001), *Vv*PnTIP2;1 (CAO45860, GSVIVT00034350001), *Vv*PnTIP2;2 (CAO23095, GSVIVT00012703001), *Vv*PnTIP3;1 (CAO62035, GSVIVT00013854001), *Vv*PnTIP4;1 (CAO44039, GSVIVT00032441001), *Vv*PnTIP5;1 (CAO42713, GSVIVT00029946001), *Vv*PnTIP5;2 (CAO70596, GSVIVT00019170001), *Vv*PnNIP1;1 (CAO48005, GSVIVT00035815001), *Vv*PnNIP2;1 (CAO15462, GSVIVT00011149001), *Vv*PnNIP3;1 (CAO17108, GSVIVT00022377001), *Vv*PnNIP4;1 (CAO70192, GSVIVT00007127001), *Vv*PnNIP4;2 (CAO43338, GSVIVT00003903001), *Vv*PnNIP5;1 (CAO62847, GSVIVT00000446001), *Vv*PnNIP6;1 (CAO45476, GSVIVT00033750001), *Vv*PnNIP7;1 (CAO71103, GSVIVT00019910001), *Vv*PnSIP1;1 (CAO23510, GSVIVT00025504001) and *Vv*PnSIP2;1 (CAO18284, GSVIVT00023346001) (for Pinot noir cultivar sequences) and *Vv*TnPIP1;1 (HQ913643), *Vv*TnPIP1;4 (KJ697714), *Vv*TnPIP2;1 (KJ697715), *Vv*TnPIP2;2 (HQ913642), *Vv*TnPIP2;3 (KJ697716), *Vv*TnTIP1;1 (KJ697717), *Vv*TnTIP2;1 (HQ913640), *Vv*TnTIP2;2 (KJ697718) and *Vv*TnTIP4;1 (KJ697719) (for Touriga nacional cultivar sequences).

Specific primers designed from the sequences of aquaporins of *Vitis vinifera* (cv. Pinot Noir) were able to amplify six full-length cDNAs of putative aquaporins from *V. vinifera* (cv. Touriga nacional). The predicted amino acid sequences were aligned and compared with the Pinot noir variety (Figure S1), all of them shared high identity (>96%) in both varieties. As expected, the six amino acids sequences corresponding to the cDNAs cloned from *V. vinifera* cv. Touriga nacional in this study were clearly identified in the genome of Pinot noir variety and three of them were clustered in their subsequent groups of PIPs, while other three in TIPs subfamilies (Figure 1). Based on BLASTN (http://blast.ncbi.nlm.nih.gov/Blast.cgi) alignment, sequences were classified as *PIP1;4* (858 bp), *PIP2;1* (852 bp), *PIP2;3* (861 bp) and *TIP1;1* (753 bp), *TIP2;2* (750 bp), *TIP4;1* (759 bp) and their encoded proteins were named as *Vv*TnPIP1;4 (286 aa), *Vv*TnPIP2;1 (284 aa), *Vv*TnPIP2;3 (287 aa) and *Vv*TnTIP1;1 (251 aa), *Vv*TnTIP2;2 (250 aa), *Vv*TnTIP4;1 (253 aa) following the nomenclature proposed for plant aquaporins [30]. *Vv*TnPIP1;4 has 96.5% identity with nine substitution of amino acids (Figure S1A), while *Vv*TnPIP2;1 is 98.6% identical with difference at four positions (Figure S1B). *Vv*TnTIP2;2 shares 99.2% identity of amino acids with substitution at two positions (Figure S1C), similarly *Vv*TnTIP1;1 has 99.6% identity with substitution at only one amino acid position (Figure S1D). On the other hand, *Vv*TnPIP2;3 and *Vv*TnTIP4;1 were 100% identical to their respective proteins of the other variety (Figures S1E and S1F, respectively).

**Figure 2:**
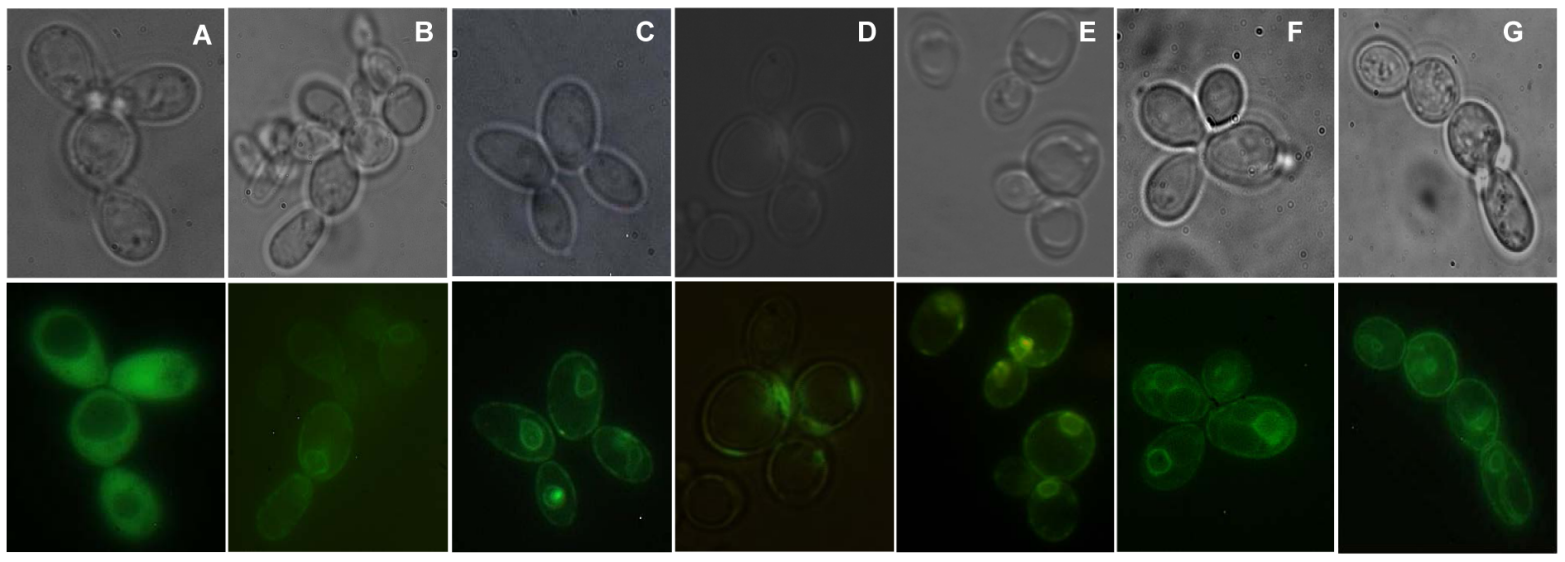
Localization of GFP-tagged aquaporins from *V. Vinifera* expressed in *S. cerevisiae* strains. Cytosolic GFP localization in (A) control cells (transformed with empty plasmid pUG35) and in the membrane of cells expressing (B) *Vv*TnPIP1;4, (C) *Vv*TnPIP2;1, (D) *Vv*TnPIP2;3, (E) *Vv*TnTIP1;1, (F) *Vv*TnTIP2;2, (G) *Vv*TnTIP4;1. Images were taken under phase contrast (upper panel) and fluorescence (lower panel) microscopy.

Expression and localization of these cloned proteins in selected yeast transformants were verified by GFP tagging under fluorescent microscopy. Most of the GFP-tagged aquaporins were localized in the plasma membrane of *S. cerevisiae* (Figure 2). Besides plasma membrane, some of the GFP-tagged proteins were also observed retained inside the cell, probably in the endoplasmic reticulum or vesicles of the secretory pathway.

### Topology prediction and analysis of conserved sequences of *Vitis vinifera* aquaporins

**Figure 3:**
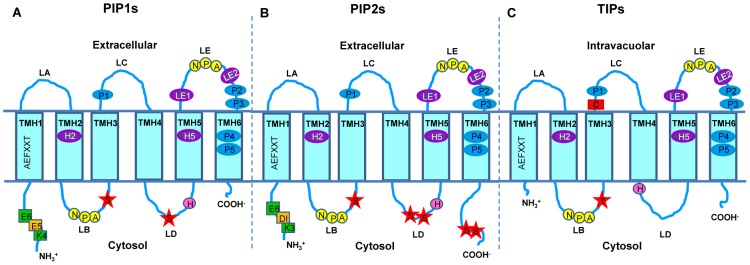
Schematic representation of predicted topology of *V. vinifera* aquaporins (PIP1s, PIP2s and TIPs) obtained in this study. These aquaporins consist of six transmembrane domains (TMH1–TMH6), five connecting loops (LA–LE) and N- and C-terminal extremities. The conserved NPA motifs (filter 1) are shown in yellow circles, while residues for ar/R constriction (filter 2) and P1–P5 positions for selectivity of atypical substrates (according to [4]) are shown in purple and blue ovals, respectively. Putative phosphorylation sites are marked as red stars, pH regulation sites are shown as pink rounds, green and yellow rectangles represent methylation and sorting signals. (A) PIP1s subfamily consisting of longer N-terminal with methylation and sorting signals, two putative phosphorylation sites in loop B and D, conserved His in loop D for pH regulation site and shorter C-terminal. (B) PIP2s subfamily having slightly shorter N-terminal with methylation and sorting signals conserved His in loop D for pH regulation, four Ser for putative phosphorylation: one in loop B, two in loop D and two in long C-terminal. (C) TIPs with very short N-terminal without sorting or methylation signals, only one conserved Thr residue instead of Ser residue for putative phosphorylation in loop B, Cys residue (red rectangle) in TMH4 for mercury sensitivity and His residue in loop D for pH regulation.

The hypothetical transmembrane topology of deduced amino acid sequences revealed that, as expected, all cloned aquaporins exhibit typical features common to orthodox plant aquaporins (Figure 3): (i) six transmembrane-spanning hydrophobic α-helices (TMH1–TMH6) connected with five alternating extracellular and intracellular loops (LA-LE), (ii) intracellular facing amino and carboxyl terminals, (iii) two highly conserved NPA (Asn-Pro-Ala) motifs on LB and LE loops important for configuration of aqueous pore in aquaporins [31], and (iv) a Serine residue near the second NPA motif (GXXXNPAR(S/D)XG), specific for water transport [32], [33].

Besides the general characteristics of aquaporins, special features attributed to the PIP aquaporin family and subfamilies (PIP1s and PIP2s) of *Vitis* aquaporins were found, such as: (i) longer N-terminal in *Vv*TnPIP1;4 (PIP1 subfamily) than *Vv*TnPIP2;1 and *Vv*TnPIP2;3 (PIP2 subfamily) (Figures 3 and S2A), (ii) longer C-terminal in *Vv*TnPIP2;1 and *Vv*TnPIP2;3 than *Vv*TnPIP1;4 [12], [34] (Figures 3 and S2B), (iii) methylation site (K3/4 and E6) [35] and diacidic motif (D/E-I/X-E/D) [36] at N-terminal (*Vv*TnPIP1;4, *Vv*TnPIP2;1 and *Vv*TnPIP2;3) (Figures 3 and S3A), (iv) His residue for pH sensitivity in loop D (*Vv*TnPIP1;4-H^206^, *Vv*TnPIP2;1-H^196^, *Vv*TnPIP2;3-H^199^) [37] (Figures 3 and S4B), (v) multiple conserved Ser residues for phosphorylation in loop B, loop D and C-terminal positions, such as 1) in loop B (*Vv*TnPIP1;4-S^128^, *Vv*TnPIP2;1-S^118^ and *Vv*TnPIP2;3-S^121^) (Figures 3 and S4A), 2) in loop D two positions of conserved Ser in consensus sequence N/SARDSHVP, in which the first Ser is present only in *Vv*TnPIP2;1 (S^191^) [38], while second Ser is present in all cloned PIPs (*Vv*TnPIP1;4-S^205^, *Vv*TnPIP2;1-S^195^ and *Vv*TnPIP2;3-S^198^) [39] (Figures 3 and S4B), 3) at C-terminal, consensus phosphorylation site (Lys-x-x-x-Ser-x-Arg) is conserved only in PIP2 subfamily members (*Vv*TnPIP2;1-S^277^ and S^280^, *Vv*TnPIP2;3-S^280^ and S^283^) [40], [41] (Figures 3 and S4C).

Distinct characteristics found in cloned TIPs were: (i) shorter N-terminals than PIPs (Figures 3 and S2A) (ii) slightly longer C-terminal than PIP1s, but shorter than PIP2s subfamily members (Figures 3 and S2B) [34] (iii) Cys residue for mercury sensitive site (*Vv*TnTIP1;1-C^118^, *Vv*TnTIP2;2-C^116^ and *Vv*TnTIP4;1- C^113^) [42] (Figures 3 and S5A), (iv) His residue for pH sensitive site (*Vv*TnTIP2;2-H^131^ and *Vv*TnTIP4;1-H^128^) (Figures 3 and S5B) [17]. (viii) Thr residue for putative phosphorylation site (*Vv*TnTIP1;1-T^99^, *Vv*TnTIP2;2-T^97^ and *Vv*TnTIP4;1-T^94^) (Figures 3 and S4A) [40], [43].

### Presence of signature sequences for transport of substrates other than water

Signature sequences for transport of atypical substrates in all cloned aquaporins were explored according to [4] (Table 1), in particular the ar/R (aromatic/Arginine) constriction (filter 2) that consists of four residues (one in the second and one in the fifth helix (TMH2 and TMH5) and two in the fifth loop (LE1 and LE2 or R)) and P1–P5 positions (P1 in the third loop (LC), P2 and P3 in the fifth loop (LE), P4 and P5 in sixth helix (TMH6)) (Figure 3). The analysis showed the presence of signature sequences deciphered for boron (filter 2: F/A/G-H/I/S-T/G/A-R and P1–P5 residues: Q/F/I-S/T-A-F/Y-W/L) (Figure S6A) and CO_2_ (filter 2: F-H-T-R and P1–P5 residues: Q/M-S-A-F-W) (Figure S6B) transport in all cloned PIPs: *Vv*TnPIP1;4 (filter 2: F^94^-H^223^-T^232^-R^238^ and P1–P5: Q^154^-S^239^-A^243^-F^258^-W^259^), *Vv*TnPIP2;1 (filter 2: F^84^-H^213^-T^222^-R^228^ and P1–P5: Q^144^-S^229^-A^233^-F^248^-W^249^) and *Vv*TnPIP2;3 (filter 2: F^87^-H^216^-T^225^-R^231^ and P1–P5: Q^147^-S^232^-A^236^-F^251^-W^252^) (Table 1). Whereas, sequences for ammonia transport (filter 2: H/W-I/V-G/A-R and P1–P5 residues: T/F-S-A-Y-W/L) (Figure S7) were found in all the TIPs: *Vv*TnTIP1;1 (filter 2: H^65^-I^187^-A^196^-V^202^ and P1–P5 residues: T^125^-S^203^-A^207^-V^219^-W^220^), *Vv*TnTIP2;2 (filter 2: H^63^-I^185^-G^194^-R^20^0 and P1–P5 residues: T^123^-S^201^-A^205^-V^217^-W^218^ and *Vv*TnTIP4;1 (filter 2: H^60^-V^183^-A^192^-R^198^ and P1–P5 residues: T^120^-S^199^-A^203^-V^215^-W^216^) (Table 1). On the other hand, all cloned aquaporins exhibited the signature sequences for H_2_O_2_ transport (filter 2: H/F/W-I/H/V-A/T/G-R/V and P1–P5 residues: T/Q/F-S/A-A-Y/F-W/I) (Figure S8A) and urea transport (filter 2: F/H/G/A/N-I/H/S/V-T/A/G-R/V and P1–P5 residues: M/T/Q/L/F/V/I-S/A/T-A-F/Y-W/F/L) (Figure S8B) (Table 1).

**Table 1:** Specific amino acid residues for atypical substrates at ar/R constrictions and P1–P5 positions (based on [4]).

Aquaporins	Putative substrates	ar/R constrictions (filter 2)				P1–P5 positions				
		TMH2	TMH5	LE1	LE2	P1	P2	P3	P4	P5
*Vv*TnPIP1;4	Boron, CO_2_, H_2_O_2_, urea	F^94^	H^223^	T^232^	R^238^	Q^154^	S^239^	A^243^	F^258^	W^259^
*Vv*TnPIP2;1	Boron, CO_2_, H_2_O_2_, urea	F^84^	H^213^	T^222^	R^228^	Q^144^	S^229^	A^233^	F^248^	W^249^
*Vv*TnPIP2;3	Boron, CO_2_, H_2_O_2_, urea	F^87^	H^216^	T^225^	R^231^	Q^147^	S^232^	A^236^	F^251^	W^252^
*Vv*TnTIP1;1	Urea, NH_3_, H_2_O_2_	H^65^	I^187^	A^196^	V^202^	T^125^	S^203^	A^207^	V^219^	W^220^
*Vv*TnTIP2;2	Urea, NH_3_, H_2_O_2_	H^63^	I^185^	G^194^	R^200^	T^123^	S^201^	A^205^	V^217^	W^218^
*Vv*TnTIP4;1	Urea, NH_3_, H_2_O_2_	H^60^	V^183^	A^192^	R^198^	T^120^	S^199^	A^203^	V^215^	W^216^

### Functional characterization of water transport in yeast strains expressing *V. vinifera* aquaporins

**Figure 4:**
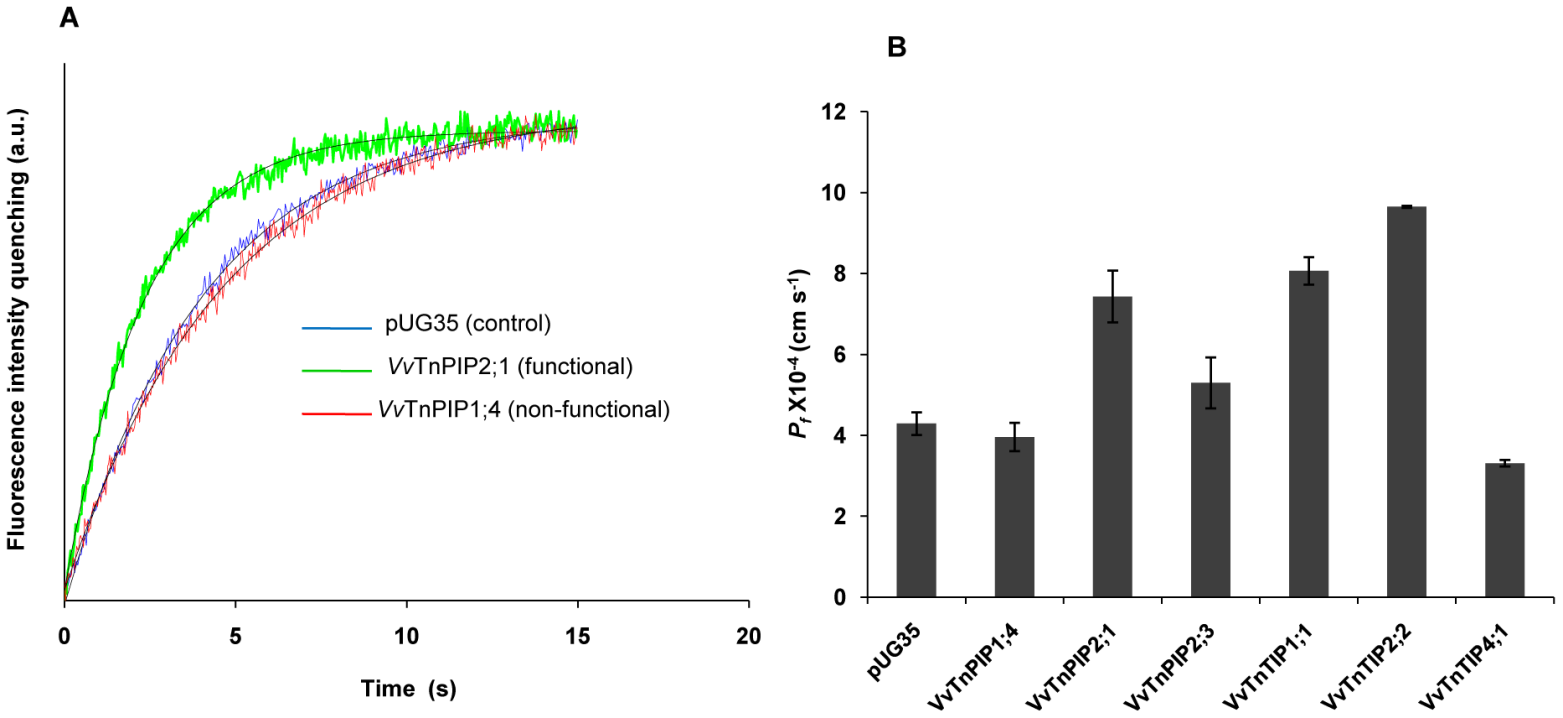
Stopped-flow assays for measurement of water transport activity of *V. vinifera* aquaporins expressed in yeast. (A) Typical traces obtained from stopped-flow spectroscopy after hyperosmotic shock. Presented signals are illustrative of ten traces at 23°C obtained from strains expressing functional (*Vv*TnPIP2;1) and non-functional (*Vv*TnTIP4;1) aquaporins for water transport, and control (transformed with empty plasmid pUG35). (B) Water permeability coefficients (*P_f_* at 23°C) of *Vv*TnPIP2;1, *Vv*TnTIP1;1 and *Vv*TnTIP2;2 were higher than control strain. Expression of *Vv*TnPIP1;4, *Vv*TnPIP2;3 and *Vv*TnTIP4;1 did not increase water permeability in yeast cells. Data are mean ± SD of three independent experiments with at least ten traces.

Water transport activity of heterologously expressed *Vitis* aquaporins in yeast strains was assayed by stopped-flow fluorescence spectroscopy. The shrinking rate of yeast cells upon a sorbitol hyperosmotic shock was monitored as change in fluorescence signals (Figure 4A). The strain with empty plasmid (pUG35), devoid of water channels, was used as control (*P_f_* = 4.30±0.28×10^−4^ cm s^−1^). Water permeability (*P_f_*) of yeast cells was not affected by the expression of *Vv*TnPIP1;4 (4.0±0.35×10^−4^ cm s^−1^), *Vv*TnPIP2;3 (5.30±0.63×10^−4^ cm s^−1^) and *Vv*TnTIP4;1 (3.3±0.08×10^−4^ cm s^−1^) (Table 2, Figure 4B). Thus, these strains were considered as non-functional for water transport. On the other hand, heterologous expression of *Vv*TnPIP2;1 (7.43±0.64×10^−4^ cm s^−1^), *Vv*TnTIP1;1 (8.06±0.34×10^−4^ cm s^−1^) and *Vv*TnTIP2;2 (9.65±0.026×10^−4^ cm s^−1^) (Table 2, Figure 4B) led to 73%, 88% and 125%, respectively, higher water permeability and were stated as functional for water transport. Among all cloned *Vitis* aquaporins, TIPs (*Vv*TnTIP1;1, *Vv*TnTIP2;2) exhibited higher water conductivity as compared to *Vv*TnPIP2;1.

**Table 2:** Water transport activity in *V. vinifera* aquaporins expressed in *S. cerevisiae*.

Yeast strains expressing *Vitis* aquaporins	Permeability coefficient (*P_f_*) (cm s^−1^)×10^−4^	Activation energy (*Ea*) (kcal mol^−1^)
pUG35	4.3±0.28	14.05±0.01
*Vv*TnPIP1;4	4.0±0.35	14.74±0.62
*Vv*TnPIP2;1	7.43±0.64	10.84±0.83
*Vv*TnPIP2;3	5.30±0.63	14.53±0.55
*Vv*TnTIP1;1	8.06±0.34	8.79±0.77
*Vv*TnTIP2;2	9.65±0.03	8.77±0.62
*Vv*TnTIP4;1	3.31±0.08	14.86±0.22

Data are mean ± SD of three independent experiments with at least ten traces.

**Figure 5:**
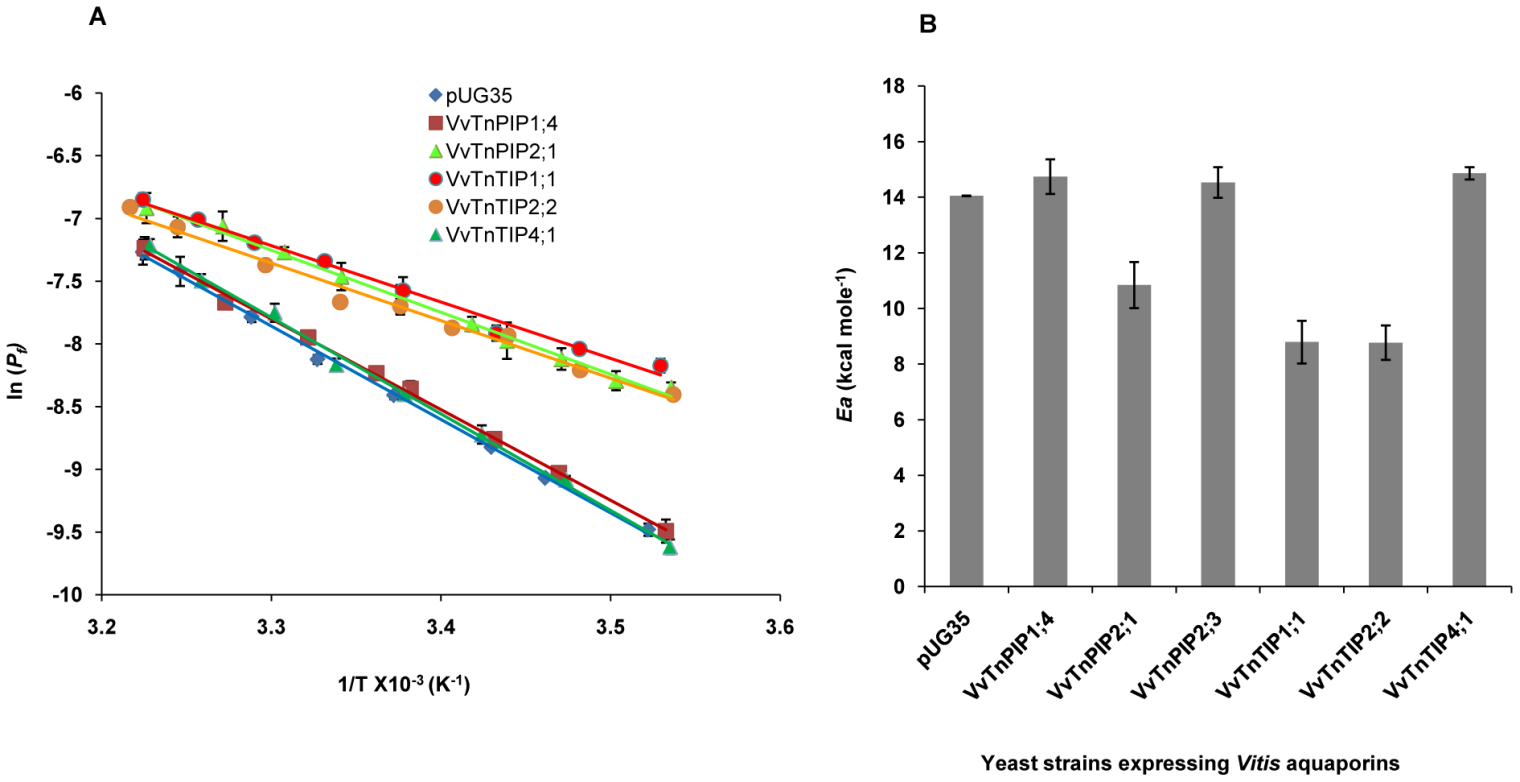
Activation energies (*Ea*) of water transport in *V. vinifera* aquaporins expressed in yeast. (A) Arrhenius plot of *P_f_* at temperature range (9–37°C), where T is temperature in Kelvin. *Ea* was evaluated from the slopes. Strains expressing *Vv*TnPIP1;4, *Vv*TnTIP4;1 and empty plasmid (pUG35) showed steeper slope, while strains expressing *Vv*TnPIP2;1, *Vv*TnTIP1;1 and *Vv*TnTIP2;2 exhibited shallow slope. (B) Calculated *Ea* from the slope showed that *Vv*TnPIP2;1, *Vv*TnTIP1;1 and *Vv*TnTIP2;2 expressing strains exhibited lower *Ea*, while *Vv*TnPIP1;4, *Vv*TnPIP2;3 and *Vv*TnTIP4;1 expressing strains showed were almost equal to control strain (pUG35). Data are mean ± SD of three independent experiments with at least five traces (ten traces in case of *P_f_* at 23°C) at each temperature.

To evaluate the activation energy for water transport, *P_f_* values of all yeast strains were analyzed in a range of temperatures (9–37°C) and Arrhenius plots were drawn (Figure 5A). As expected, transport of water was strongly dependent on temperature in control yeast strain, as reflected by higher activation energy *Ea* (14.05±0.01 kcal mol^−1^) (Figure 5B and Table 2). Arrhenius plots for *Vv*TnPIP1;4, *Vv*TnPIP2;3 and *Vv*TnTIP4;1 expressing yeast strains exhibited parallel steep slopes, overlapped with control strain (Figure 5A) and almost similar activation energies (14.74±0.62, 14.53±0.55, 14.86±0.22 kcal mol^−1^, respectively) (Figure 5B and Table 2). On the other hand, expression of *Vv*TnPIP2;1, *Vv*TnTIP1;1 and *Vv*TnTIP2;2 led to drastic reduction in the activation energies to 10.84±0.83, 8.8±0.77 and 8.77±0.62 kcal mol^−1^, respectively (Figure 5B and Table 2), demonstrating that transport of water was majorly mediated by aquaporins in these yeast strains.

**Figure 6:**
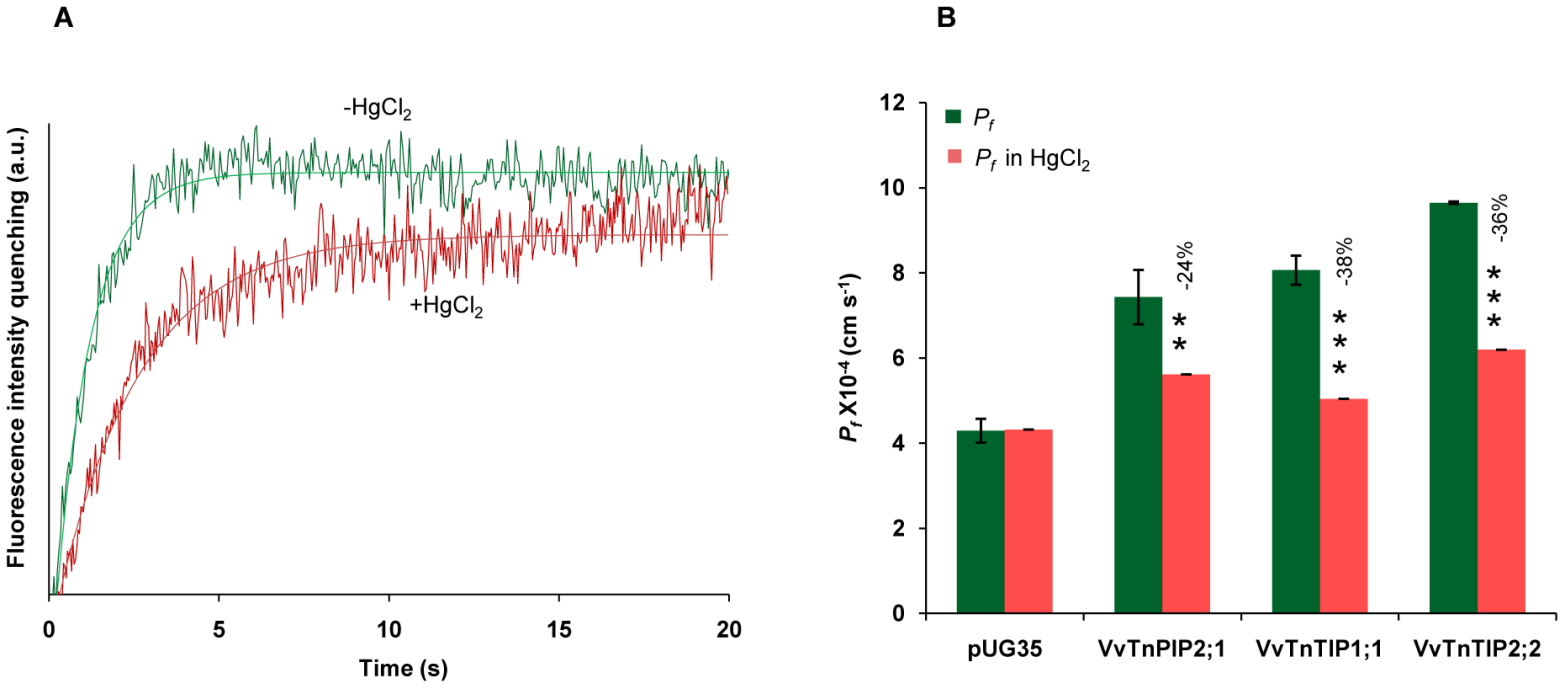
Inhibition of water permeability by mercury chloride (HgCl_2_) in yeast strains expressing *V. vinifera* aquaporins. (A) Inhibition of water permeability in yeast cells expressing functional aquaporin (*Vv*TnTIP2;2) by HgCl_2_. The green signal represents water permeability without HgCl_2_ and the red signal shows the lower permeability when cells were incubated with 0.5 mM HgCl_2_ for 15 minutes prior to osmotic shock. (B) Water permeability coefficients (*P_f_* at 23°C) of *Vv*TnPIP2;1, *Vv*TnTIP1;1 and *Vv*TnTIP2;2 were reduced by 0.5 mM HgCl_2_. Data are mean ± SD of three independent experiments with at least five traces. The data were analyzed by *t-test* and asterisks above bars indicate statistically significant differences, where **p<0.01, ***p<0.001.

To examine the dose and time dependent effects of mercurial inhibition, yeast strains expressing functional *Vitis* aquaporins (*Vv*TnPIP2;1, *Vv*TnTIP1;1 and *Vv*TnTIP2;2) as well as the control strain were pretreated with various concentrations (0.5 and 1.0 mM) of HgCl_2_ for 15 to 30 minutes at room temperature, before hyperosmotic shock. Figure 6A compares the stopped-flow signals of yeast cells expressing *Vv*TnTIP2;2, obtained after hyperosmotic shock either in the absence or presence of mercury chloride (0.5 mM) after 15 minutes of incubation. It is clear from the signals that HgCl_2_ reduced the shrinking rates of the cells. All the functional aquaporins tested were mercury sensitive. TIPs were more sensitive than PIP. *P_f_* was markedly decreased when cells were incubated with 0.5 mM HgCl_2_ for 15 minutes before the osmotic shock. *P_f_* of *Vv*TnPIP2;1 expressing strain was reduced by 24% while *Vv*TnTIP1;1 and *Vv*TnTIP2;2 expressing strains exhibited 38% and 36% reduction (Figure 6B). Water transport in control cells was not affected by 0.5 mM HgCl_2_, even after 30 minutes of incubation, while higher concentration (1 mM) reduced the *P_f_* of all yeast strains including the control strain, indicating a general toxicity at this concentration (data not shown).

### Growth assays under osmotic stress and in the presence of atypical substrates

Expression of *Vitis* aquaporins in *S. cerevisiae* was not deleterious to yeast cells, since neither the specific growth rate nor the final biomass of the yeast were affected during growth in standard YNB media. Accordingly, all the strains showed approximately 3.5 hours of doubling time and grew up to similar final biomass in liquid YNB media (data not shown).

#### Expression of Vitis aquaporins affects the growth of yeast cells under osmotic stress

**Figure 7:**
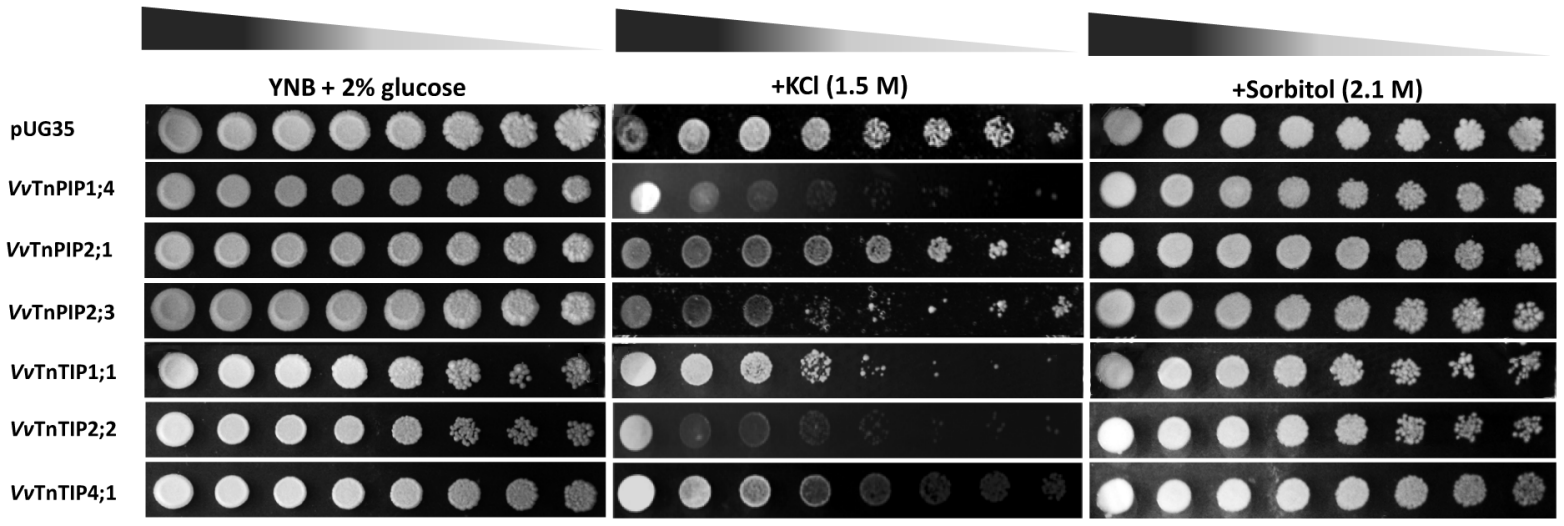
Growth assays of *S. cerevisiae* strains expressing *V. vinifera* aquaporins under osmotic stress. Hyperosmotic stress was exerted by osmo-equivalent concentration of either KCl or sorbitol. Yeast strain transformed with empty pUG35 plasmid was used as control (pUG35). Yeast suspensions were spotted in 10-fold dilution on solid YNB plates without or with 1.5 M KCl or 2.1 M sorbitol. Growth was recorded after two weeks at 28°C. Photographs shown are representative of three independent experiments with consistent results.

Osmotic tolerance/susceptibility of heterologously expressed *Vitis* aquaporins was examined in the presence of NaCl, KCl and sorbitol. When exposed to hyperosmotic stress, yeast cells expressing *Vitis* aquaporins exhibited decreased growth to a variable degree, depending on the types of aquaporins expressed (Figure 7). Growth of all yeast strains (including empty plasmid strain) was equally inhibited in the presence of NaCl, even at lower concentration (0.5 M) (data not shown). At the highest concentration of KCl (1.5 M), cells expressing *Vv*TnTIP4;1 were least inhibited by KCl, while growth of *Vv*TnTIP2;2 and *Vv*TnPIP1;4 expressing cells displayed a clear osmosensitive phenotype followed by *Vv*TnTIP1;1, *Vv*TnPIP2;3 and *Vv*TnPIP2;1 (Figure 7). Growth of these strains was reduced under osmoequivalent concentration of sorbitol (2.1 M), but did not show any clear phenotype (Figure 7).

#### Expression of *V. vinifera* aquaporins increases the sensitivity of yeast to H_2_O_2_ and boron

Ability of *Vitis* aquaporins to facilitate the transport of H_2_O_2_ and boron was examined by drop test assays in the presence of these substrates on solid YNB (pH 5.0). Impaired growth of yeast strains due to the expression *Vitis* aquaporins was tested in the presence of these substrates.

**Figure 8:**
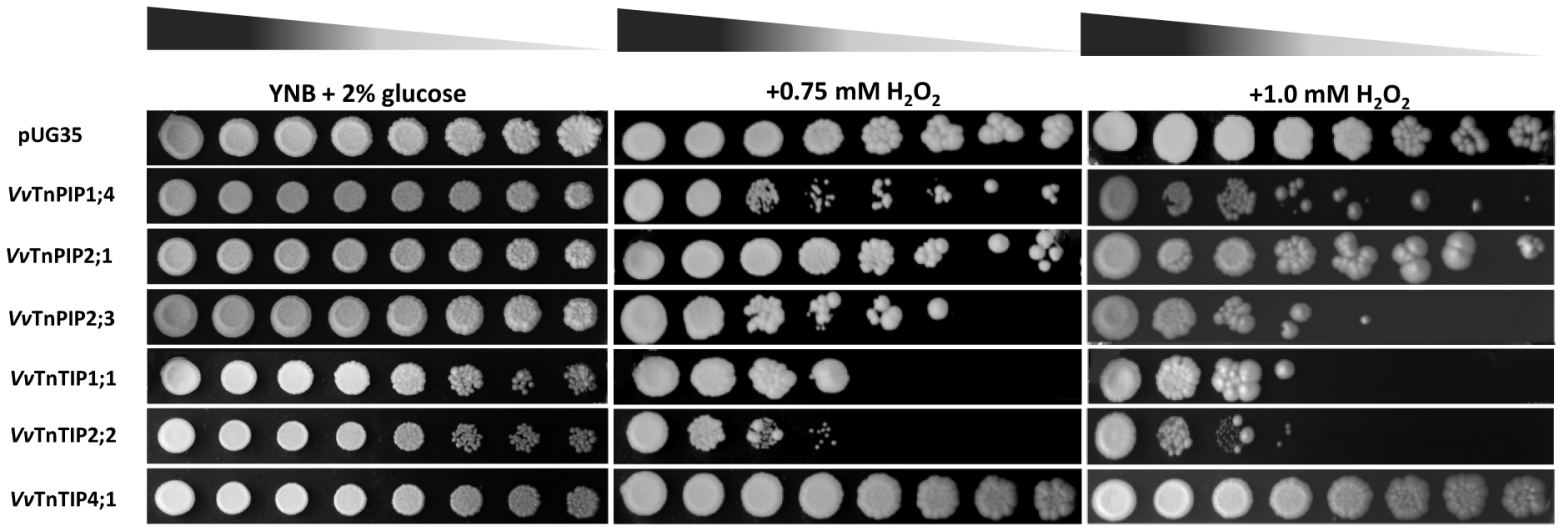
Growth assays of *S. cerevisiae* strains expressing *V. vinifera* aquaporins on H_2_O_2_ containing minimal media. Yeast strain transformed with empty pUG35 plasmid was used as control (pUG35). Yeast suspensions were spotted in 10-fold dilution on solid YNB plates without or with 0.75 mM and 1.0 mM H_2_O_2_. Growth was recorded after two weeks at 28°C. Photographs shown are representative of three independent experiments with consistent results.

Growth of *Vitis* aquaporins (*Vv*TnPIP1;4, *Vv*TnPIP2;1, *Vv*TnPIP2;3, *Vv*TnTIP1;1 and *Vv*TnTIP2;2) expressing strains was affected by externally supplied H_2_O_2_ in a dose dependent manner (Figure 8). Impaired growth of *Vitis* aquaporins expressing strains was observed at higher concentration of H_2_O_2_ (0.75 mM) (Figure 8), while the control strain was able to grow up to the last dilution at 1.0 mM H_2_O_2_. Growth of the strain expressing *Vv*TnTIP2;2 was most severely inhibited, followed by *Vv*TnTIP1;1, *Vv*TnPIP2;3, *Vv*TnPIP1;4 and *Vv*TnPIP2;1 expressing strains (Figure 8)_._ Cells expressing *Vv*TnTIP4;1 were least sensitive toward H_2_O_2_ and exhibited similar growth as the control strain.

**Figure 9:**
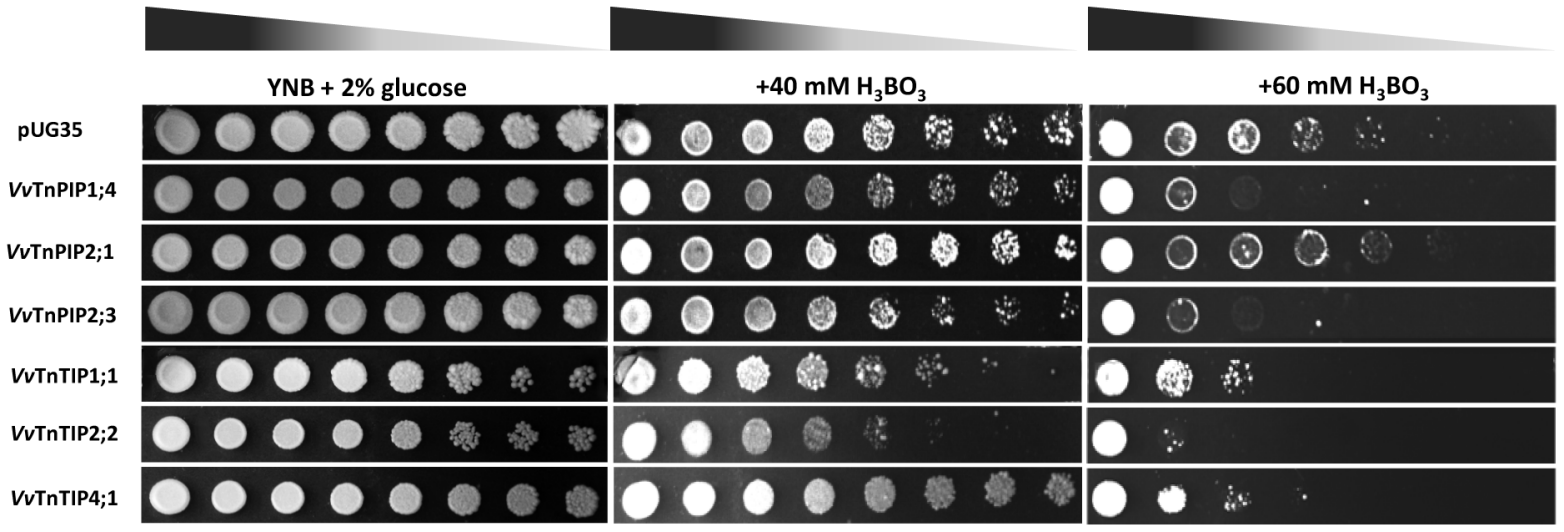
Growth assays of *S. cerevisiae* strains expressing *V. Vinifera* aquaporins on minimal medium containing boric acid. Yeast strain transformed with empty pUG35 plasmid was the control (pUG35). Yeast suspensions were spotted in 10-fold dilution on solid YNB plates without or with 40 mM and 60 mM boric acid. Growth was recorded after two weeks at 28°C. Photographs shown are representative of three independent experiments with consistent results.

The ability of *Vitis* aquaporins to facilitate boron transport was tested in the same way as H_2_O_2_. Yeast transformants were spotted on YNB plates containing various concentrations of boron as boric acid (Figure 9)_._ The exposure to boric acid (40 mM) caused reduced growth of strains expressing *Vitis* aquaporin. Interestingly, at 40 mM boric acid, cells transformed with *Vv*TnTIP4;1 grew better in comparison to control strain, but these cells showed clear sensitivity toward boric acid when exposed to higher concentration (60 mM) of boric acid. Heterologous expression of all cloned *Vitis* aquaporins, except *Vv*TnPIP2;1, showed increased sensitivity to 60 mM boric acid. *Vv*TnPIP2;1 expressing strain was the most tolerant to externally supplied boric acid and was able to grow as the control strain. Growth of *Vv*TnTIP2;2 expressing strain was severely inhibited by boric acid exposure (Figure 9)_._

We also performed the growth assays of yeast strains expressing *Vitis* aquaporins in the presence of ammonia and urea, to explore their possible transport by aquaporins. We observed that growth of yeast strains was not affected by these substrates at any tested concentrations, since no distinct growth phenotypes were observed (results not shown).

## Discussion

In this study we have cloned and characterized three plasma membrane (PIPs) and three tonoplast (TIPs) aquaporins from *Vitis vinifera* cv. Touriga nacional, an important Portuguese cultivar, widely believed to produce the finest red wines of Portugal.

All the cloned full length aquaporin genes from *V. vinifera* (cv. Touriga nacional) encoding three PIPs (*Vv*TnPIP1;4, *Vv*TnPIP2;1 and *Vv*TnPIP2;3) and TIPs (*Vv*TnTIP1;1, *Vv*TnTIP2;2 and *Vv*TnTIP4;1) proteins showed obvious similarity with the same proteins of the database variety of *V. vinifera* (cv. Pinot noir). Their plasma membrane localization was confirmed by GFP-tagging, although we observed a partial retention in intracellular structures, probably in endoplasmic reticulum or in vesicles of secretory pathway (reviewed by [6]). Several reports suggest that a fraction of intracellular plant aquaporins (TIPs) is generally “miss-targeted” to the plasma membrane of heterologous models, which enabled the researchers to measure their water transport activity in *Xenopus* and yeast (reviewed by [44]).

Their sequence analysis for topological prediction revealed all the characteristics of typical aquaporin subfamilies. Specific residues for transport of ammonia (filter 2: H/W-I/V-G/A-R and P1–P5 residues: T/F-S-A-Y-W/L), boron (filter 2: F/A/G-H/I/S-T/G/A-R and P1–P5 residues: Q/F/I-S/T-A-F/Y-W/L), CO_2_ (filter 2: F-H-T-R and P1–P5 residues: Q/M-S-A-F-W), H_2_O_2_ (filter 2: H/F/W-I/H/V-A/T/G-R/V and P1–P5 residues: T/Q/F-S/A-A-Y/F-W/I) and urea (filter 2: F/H/G/A/N-I/H/S/V-T/A/G-R/V and P1–P5 residues: M/T/Q/L/F/V/I-S/A/T-A-F/Y-W/F/L) were present at ar/R constriction and P1–P5 positions [4] of the cloned *Vitis* aquaporins, depending on their subfamilies (Table 1).

Expression of functional aquaporins *Vv*TnPIP2;1, *Vv*TnTIP1;1 and *Vv*TnTIP2;2 increased the water permeability coefficient (*P_f_*) and reduced the activation energy (*Ea*) required for water transport in yeast cells, while expression of non-functional aquaporins *Vv*TnPIP1;4, *Vv*TnPIP2;3 and *Vv*TnTIP4;1 expression exhibited higher *Ea* and did not affect *P_f_*. The obtained higher *Ea* and lower *P_f_* can not be correlated with their lower plasma membrane expression since all the functional aquaporins were also partially retained at various subcellular regions and, on the other hand, one of the aquaporins (*Vv*TnTIP4;1) showed to be localized at the plasma membrane of *S. cerevisiae* and exhibited low water transport.

TIPs (*Vv*TnTIP1;1 and *Vv*TnTIP2;2) exhibited higher water conductivity in comparison to PIP (*Vv*TnPIP2;1). This finding is in agreement with previous reports of heterologously expressed plant aquaporins [41], [45], [46]. Higher water permeability of TIPs may be required for the central role of vacuole in osmoregulation of the cytoplasm. Constitutively higher water permeability of tonoplast aquaporins may allow the cells to use the vacuolar space to maintain the cellular integrity, in case of osmotic fluctuations caused by rapid water exchange during stress or developmental stages of plants (reviewed in [43], [45]).

All functional *Vitis* aquaporins were found to be mercury-sensitive, as their *P_f_* was reduced by mercury chloride. Many plant aquaporins have been inhibited by mercurial reagents, which bind to a conserved cysteine residue located downstream to first NPA motif, subsequently blocking the pore and shutting-down the aqueous pathway (reviewed in [3]). Notably all cloned TIPs showed the conserved cysteine residue responsible for mercury sensitivity (Figure S5A). Although PIPs (except *Vv*TnPIP2;3) did not have the conserved cysteine at the same position of alignment, they have four conserved cysteine residues in second and third transmembrane helices and one of these residues may represent the mercury sensitive site [47].

Transport of solutes of physiological significance, such as CO_2_, H_2_O_2_, boron or silicic acid is now well established and has linked aquaporins to many functions, including carbon metabolism, oxidative stress responses, and plant mineral nutrition [3]. We performed a broad screening of growth sensitivity test of all cloned *Vitis* aquaporins on H_2_O_2_ and boric acid containing plates to investigate their ability to transport these atypical substrates. The phenotypic growth variations showed that heterologous expression of all cloned *Vitis* aquaporins (except *Vv*TnTIP4;1) caused more susceptibility to externally applied H_2_O_2_, indicating their putative role in H_2_O_2_ transport. In principle, increased level of H_2_O_2_ can disturb the cellular metabolism in an unidentified manner, which may lead to reduced or impaired growth of yeast cells. A higher permeability of H_2_O_2_ by heterologously expressed aquaporins might have increased the influx of H_2_O_2_ into yeast cells and eventually triggered the cell death or growth inhibition. H_2_O_2,_ a long-lived reactive oxygen species (ROS) shares molecular properties with water, it can form hydrogen bonds and has similar permeability as water [48], [49]. Based on the sensitivity of yeast cells expressing *Vitis* aquaporins, we assume that both *Vitis* PIPs and TIPs may permeate H_2_O_2_. Similar results were previously reported in heterologously expressed *Arabidopsis* [50]–[52] and maize aquaporins [53] expressed in *S. cerevisiae*. It is noteworthy that putative amino acids for H_2_O_2_ transport were found at ar/R constriction of all cloned *Vitis* aquaporins. Besides a potential threat to biological components, H_2_O_2_ also acts as a physiologically important molecule to activate Ca^2+^ channels for NADPH oxidase activity required during root hair growth and stomatal movement [54]. Under stress, H_2_O_2_ can be compartmentalized in various intracellular organelles as well as outside of the cells in apoplastic regions [55]. Further, the extracellular accumulated H_2_O_2_ can act as a regulator of water homeostasis by triggering the PIP internalization in *Arabidopsis* roots and consequently reduces the water permeability in roots under stress [56]. The role of *Vitis* PIPs may be involved in apoplastic exclusion of excess H_2_O_2_. Our results indicate that not only plasma membrane aquaporins (PIPs), but also tonoplast aquaporins (TIPs) are involved in H_2_O_2_ transport. Results of growth assays suggest that the cells expressing TIPs were more sensitive as compared with the cells expressing PIPs. TIPs are reported to be more diverse transporters and can conduct many solutes other than water due to having relatively weak selectivity (reviewed in [52], [57]). Under elevated stress conditions, plant TIPs are suggested to transport high amounts of H_2_O_2_ into vacuoles, resulting in their further detoxification by vacuolar peroxidases using vacuole stored flavanoids as an electron donors [50]. Expression level of TIPs was found to be affected by H_2_O_2_ in tulip flowers [58] and it was suggested that TIPs triggered the ROS translocation into the vacuoles for their detoxification [57].

When exposed to H_2_O_2_, all yeast cells exhibited fluffy and larger colony morphology with rough edges (results not shown). H_2_O_2_ induces aging of the cells by releasing free radicals, leading to higher number of old cells which are bigger in size in comparison to young cells and eventually make the larger colonies with mature cells [59]. Fluffy and rough surface colonies may represent a metabolic strategy of growth under unfavorable conditions [60].

Growth assays in the presence of boric acid showed that strains expressing *Vitis* aquaporins were more sensitive in comparison to control strain. Heterologous expression of plant aquaporins either in *Xenopus laevis* oocytes or *S. cerevisiae*, suggests that besides other routes, boron can be transported through PIPs [61], [62] and NIPs [63]–[66]. NIP5;1 and NIP6;1 of *Arabidopsis thaliana* were identified as boric acid channels and were up-regulated under limited boron supply to accomplish the demand of boric acid [63], [64]. Although boron is an essential micronutrient for plants, when accumulated at higher concentration it leads to toxicity. Being a non-charged molecule, boric acid can passively diffuse across lipid bilayer, but only significantly at high concentration gradient caused by high boron supply [67]. In plants, urea (*DUR3*) and glycerol (*FPS1*) transporters were identified as boron importers, while *BOR1* and its homolog *ScBOR1* of *S. cerevisiae* were characterized as boron exporters, exporting boron and protecting the cells from boron toxicity [68]. In this work, all the yeast strains expressing *Vitis* aquaporins have the same background for passive diffusion or importers and exporters of boric acid in their native membrane. Since different levels of toxicity were detected (Figure 9), we can speculate the putative transport of boron by *Vitis* aquaporins resulting in growth inhibition. Similarly, heterologously expressed barley *Hv*NIP2;1 [66], *Hv*PIP1;3 and *Hv*PIP1;4 [62] displayed sensitive growth phenotype in the presence of boron and were identified as boron transporters. Interestingly, all PIPs exhibited the signature sequences for boron transport and showed sensitivity (except *Vv*TnPI2;1) to boric acid. On the contrary, these conserved residues were not present in TIPs, although yeast strains expressing TIPs also exhibited increased sensitivity toward boron.

Although these studies are based on the molecular analysis of ar/R regions of *Vitis* aquaporins and indirect yeast based sensitivity growth assays on these substrates, transport of H_2_O_2_ and boron by *Vitis* aquaporins can be hypothesized.

In conclusion, we have cloned six PIPs and TIPs aquaporins from grapevine (cv. Touriga nacional) in *S. cerevisiae*, and have shown that three of them facilitate the transport of water. Reduced growth and survival of *Vitis* aquaporins expressing yeast cells in the presence of H_2_O_2_ and boron, indicates their probable role in transport of these non-aqua substrates. Additionally, expression of three aquaporins in yeast, which were found non-functional for water transport, exhibited sensitive phenotype when grown in the presence of these substrates, suggests their possible role in transport of substrates other than water. The combined use of molecular analysis of their sequences and functional investigation of growth sensitivity assays provided the initial insight on the selectivity profiles of *Vitis* aquaporins. Further studies are required to ascertain the role of *Vitis* aquaporins in transport of H_2_O_2_ and boron. Moreover, co-expression of aquaporins in *S. cerevisiae* may represent the next step for better understanding their interaction and regulation in plants.

## Supporting Information

Figure S1 **Comparison of** ***V. vinifera*** **aquaporins from Pinot noir and Touriga nacional cultivars.** Alignment of deduced amino acid sequences of *Vv*TnPIP1;4 (A), *Vv*TnPIP2;1 (B), *Vv*TnPIP2;3 (C), *Vv*TnTIP1;1 (D), *Vv*TnTIP2;2 (E), *Vv*TnTIP4;1 (F) obtained from the present study (Touriga nacional) with the database variety (Pinot noir). Accession numbers of presented protein sequences are: *Vv*PnPIP1;4 (CAO39626), *Vv*TnPIP1;4 (KJ697714), *Vv*PnPIP2;1 (CAN75442), *Vv*TnPIP2;1 (KJ697715), *Vv*PnPIP2;3 (CAO18152), *Vv*TnPIP2;3 (KJ697716), *Vv*PnTIP1;1 (CAO69259), *Vv*TnTIP1;1 (KJ697717), *Vv*PnTIP2;2 (CAO23095), *Vv*TnTIP2;2 (KJ697718), *Vv*PnTIP4;1 (CAO44039), *Vv*TnTIP4;1 (KJ697719).
(PDF)

Figure S2 **Comparison of N- (A) and C- (B) terminals of PIP1s, PIP2s and TIPs aquaporins.** Aquaporins cloned in the present study are marked with red arrows. Accession numbers of presented protein sequences are: *At*PIP2;1 (P43286), *At*-deltaTIP2 (CAB10515), *At*-gammaTIP3 (AAC62778), *At*-epsilonTIP (AAC42249), *Fa*PIP2;1 (ADJ67992), *Pv*-alphaTIP (CAA44669), *So*PIP2;1 (4JC6_N), *Vv*PnPIP1;1 (CAO41326), *Vv*TnPIP1;1 (HQ913643), *Vv*PnPIP1;4 (CAO39626), *Vv*TnPIP1;4 (KJ697714), *Vv*PnPIP2;1 (CAN75442), *Vv*TnPIP2;1 (KJ697715), *Vv*PnPIP2;2 (CAO47394), *Vv*TnPIP2;2 (HQ913642), *Vv*PnPIP2;3 (CAO18152), *Vv*TnPIP2;3 (KJ697716), *Vv*PnTIP1;1 (CAO69259), *Vv*TnTIP1;1 (KJ697717), *Vv*PnTIP2;1 (CAO45860), *Vv*TnTIP2;1 (HQ913640), *Vv*PnTIP2;2 (CAO23095), *Vv*TnTIP2;2 (KJ697718), *Vv*PnTIP4;1 (CAO44039), *Vv*TnTIP4;1 (KJ697719), *Zm*PIP2;1 (Q84RL7), *Zm*PIP2;5 (Q9XF58). *At*: *Arabidopsis thaliana*, *Fa*: *Fragaria×ananassa*, *Pv*: *Phaseolus vulgaris*, *So*: *Spinacia oleracea*, *Vv*Pn: *Vitis vinifera* cv. Pinot noir, *Vv*Tn: *V. vinifera* cv. Touriga nacional.
(PDF)

Figure S3 **Presence of methylation and sorting signal sequences (D/E-X-D/E) at N-terminal (A) and pH regulation site (His residue) for gating (B) of PIPs aquaporins.** Consensus sequences are framed and conserved residues are indicated as red triangle. Aquaporins cloned in the present study are marked with red arrows. Accession numbers of presented protein sequences are: *At*PIP2;1 (P43286), *At*PIP1;2 (Q06611), *At*PIP1;4 (Q39196), *Fa*PIP2;1 (ADJ67992), *So*PIP2;1 (4JC6_N), *Vv*PnPIP1;1 (CAO41326), *Vv*TnPIP1;1 (HQ913643), *Vv*PnPIP1;4 (CAO39626), *Vv*TnPIP1;4 (KJ697714), *Vv*PnPIP2;1 (CAN75442), *Vv*TnPIP2;1 (KJ697715), *Vv*PnPIP2;2 (CAO47394), *Vv*TnPIP2;2 (HQ913642), *Vv*PnPIP2;3 (CAO18152), *Vv*TnPIP2;3 (KJ697716), *Zm*PIP2;1 (Q84RL7), *Zm*PIP2;5 (Q9XF58), *Zm*PIP2;4 (Q9ATM6). *At*: *Arabidopsis thaliana*, *Fa*: *Fragaria×ananassa*, So: *Spinacia oleracea*, *Vv*Pn: *Vitis vinifera* cv. Pinot noir, *Vv*Tn: *V. vinifera* cv. Touriga nacional, *Zm*: *Zea mays*.
(PDF)

Figure S4 **Putative phosphorylation sites in aquaporins.** Ser residue in PIPs and Thr residue in TIPs aquaporins in loop B (A), loop D (B) and C-terminal (C) are tentative phosphorylation sites. Consensus sequences are framed and conserved residues are indicated as red triangle. Aquaporins cloned in the present study are marked with red arrows. Accession numbers of presented protein sequences are: *At*PIP1;2 (Q06611), *At*PIP1;4 (Q39196), *At*PIP2;1 (P43286), *At*-deltaTIP2 (CAB10515), *At*-gammaTIP3 (AAC62778), *At*-alphaTIP (AAC42249), *Fa*PIP2;1 (ADJ67992), *So*PIP2;1 (4JC6_N), *Vv*PnPIP1;1 (CAO41326), *Vv*TnPIP1;1 (HQ913643), *Vv*PnPIP1;4 (CAO39626), *Vv*TnPIP1;4 (KJ697714), *Vv*PnPIP2;1 (CAN75442), *Vv*TnPIP2;1 (KJ697715), *Vv*PnPIP2;2 (CAO47394), *Vv*TnPIP2;2 (HQ913642), *Vv*PnPIP2;3 (CAO18152), *Vv*TnPIP2;3 (KJ697716), *Vv*PnTIP1;1 (CAO69259), *Vv*TnTIP1;1 (KJ697717), *Vv*PnTIP2;1 (CAO45860), *Vv*TnTIP2;1 (HQ913640), *Vv*PnTIP2;2 (CAO23095), *Vv*TnTIP2;2 (KJ697718), *Vv*PnTIP4;1 (CAO44039), *Vv*TnTIP4;1 (KJ697719), *Zm*PIP2;1 (Q84RL7), *Zm*PIP2;4 (Q9ATM6), *Zm*PIP2;5 (Q9XF58). *At*: *Arabidopsis thaliana*, *Fa*: *Fragaria×ananassa*, *Pv*: *Phaseolus vulgaris*, *So*: *Spinacia oleracea*, *Vv*Pn: *Vitis vinifera* cv. Pinot noir, *Vv*Tn: *V. vinifera* cv. Touriga nacional, *Zm*: *Zea mays*.
(PDF)

Figure S5 **Mercury sensitive site and pH regulation site in TIPs.** Alignment of TIPs aquaporins for (A) mercury sensitive site (Cys residue) and (B) pH regulation site (His residue) for gating. Consensus sequences are framed and conserved residues are indicated as red triangle. TIPs cloned in the present study are marked with red arrows. Accession numbers of presented protein sequences are: *At*-deltaTIP2 CAB10515), *At*-gammaTIP3 (AAC62778), *At*-alphaTIP (AAC42249), *Pv*-alphaTIP (CAA44669), *Vv*PnTIP1;1 (CAO69259), *Vv*TnTIP1;1 (KJ697717), *Vv*PnTIP2;1 (CAO45860), *Vv*TnTIP2;1 (HQ913640), *Vv*PnTIP2;2 (CAO23095), *Vv*TnTIP2;2 (KJ697718), *Vv*PnTIP4;1 (CAO44039), *Vv*TnTIP4;1 (KJ697719). *At*: *Arabidopsis thaliana*, *Pv*: *Phaseolus vulgaris*, *Vv*Pn: *Vitis vinifera* cv. Pinot noir, *Vv*Tn: *V. Vinifera* (cv. Touriga nacional).
(PDF)

Figure S6 **Consensus sequences for transport of boron and CO_2_.** Alignment of putative amino acids of aquaporins of *V. Vinifera* (cv. Touriga nacional) obtained from present study and previous study [17] with the sequences of aquaporins reported to transport (A) boron and (B) CO_2_. ar/R constrictions and P1–P5 positions are shown to demonstrate the conserved amino acid residue. Accession numbers of presented protein sequences are: *At*PIP1;2 (Q06611), *At*NIP5;1 (NP_192776), *Hv*PIP1;3 (BAA23745), *Hv*PIP2;1 (BAA23744), *Nt*AQP1 (O24662), *Os*NIP2;1 (Q6Z2T3), *Vv*TnPIP1;1 (HQ913643), *Vv*TnPIP1;4 (KJ697714), *Vv*TnPIP2;1 (KJ697715), *Vv*TnPIP2;2 (HQ913642), *Vv*TnPIP2;3 (KJ697716), *Vv*TnTIP1;1 (KJ697717), *Vv*TnTIP2;1 (HQ913640), *Vv*TnTIP2;2 (KJ697718), *Vv*TnTIP4;1 (KJ697719), *Zm*PIP1;1 (Q41870). *At*: *Arabidopsis thaliana*, *Hv*: *Hordeum vulgare*, *Vv*Tn: *V. vinifera* (cv. Touriga nacional), *Zm*: *Zea mays*.
(PDF)

Figure S7 **Consensus sequences for transport of ammonia.** Alignment of putative amino acids of aquaporins of *V. Vinifera* (cv. Touriga nacional) obtained from present study and previous study [17] with sequences of aquaporins reported to transport ammonia. ar/R constrictions and P1–P5 positions are shown to demonstrate the conserved amino acid residue. Accession numbers of presented protein sequences are: *At*TIP2;1 (Q41951), *At*TIP2;3 (Q9FGL2), *Gm*NOD26 (P08995), *Ta*TIP2;1 (AAS19468),*Vv*TnPIP1;1 (HQ913643), *Vv*TnPIP1;4 (KJ697714), *Vv*TnPIP2;1 (KJ697715), *Vv*TnPIP2;2 (HQ913642), *Vv*TnPIP2;3 (KJ697716), *Vv*TnTIP1;1 (KJ697717), *Vv*TnTIP2;1 (HQ913640), *Vv*TnTIP2;2 (KJ697718), *Vv*TnTIP4;1 (KJ697719). *At*: *Arabidopsis thaliana*, *Gm*: *Glycine max*, *Ta*: *Triticum aestivum*, *Vv*Tn: *V. Vinifera* (cv. Touriga nacional).
(PDF)

Figure S8 **Consensus sequences for transport of H_2_O_2_ and urea.** Alignment of putative amino acids of aquaporins of *V. Vinifera* (cv. Touriga nacional) obtained from present study and previous study [17] with sequences of aquaporins reported to transport (A) H_2_O_2_ and (B) urea. ar/R constrictions and P1–P5 positions are shown to demonstrate the conserved amino acid residue. Accession numbers of presented protein sequences are: *At*TIP1;1 (P25818), *At*TIP1;2 (Q41963), *At*TIP1;3 (NP_192056), *At*TIP2;1 (Q41951), *At*TIP2;3 (Q9FGL2), *At*PIP2;4 (Q9FF53), *Cp*NIP1 (CAD67694), *Nt*AQP1 (O24662), *Nt*TIPa (Q9XG70), *Os*NIP2;1 (Q6Z2T3), *Vv*TnPIP1;1 (HQ913643), *Vv*TnPIP1;4 (KJ697714), *Vv*TnPIP2;1 (KJ697715), *Vv*TnPIP2;2 (HQ913642), *Vv*TnPIP2;3 (KJ697716), *Vv*TnTIP1;1 (KJ697717), *Vv*TnTIP2;1 (HQ913640), *Vv*TnTIP2;2 (KJ697718), *Vv*TnTIP4;1 (KJ697719), *Zm*PIP1;5 (Q9AR14). *At*: *Arabidopsis thaliana*, *Cp*: *Cucurbita pepo*, *Nt*: *Nicotiana tabacum*, *Os*: *Oryza sativa*, *Vv*Tn: *Vitis vinifera* (cv. Touriga nacional), *Zm*: *Zea mays*.
(PDF)

Table S1 **Primers used in this work (restriction sites are underlined).**
(PDF)
