# Supplementary material for: Exploring Three PIPs and Three TIPs of Grapevine for Transport of Water and Atypical Substrates through Heterologous Expression in aqy-null Yeast
Source: PLoS One. 2014 Aug 11;9(8):e102087. doi: 10.1371/journal.pone.0102087 (PMC4128642; doi:10.1371/journal.pone.0102087)
Supplement: Figure S1 — Comparison of V. vinifera aquaporins from Pinot noir and Touriga nacional cultivars. Alignment of deduced amino acid sequences of VvTnPIP1;4 (A), VvTnPIP2;1 (B), VvTnPIP2;3 (C), VvTnTIP1;1 (D), VvTnTIP2;2 (E), VvTnTIP4;1 (F) obtained from the present study (Touriga nacional) with the database variety (Pinot noir). Accession numbers of presented protein sequences are: VvPnPIP1;4 (CAO39626), VvTnPIP1;4 (KJ697714), VvPnPIP2;1 (CAN75442), VvTnPIP2;1 (KJ697715), VvPnPIP2;3 (CAO18152), VvTnPIP2;3 (KJ697716), VvPnTIP1;1 (CAO69259), VvTnTIP1;1 (KJ697717), VvPnTIP2;2 (CAO23095), VvTnTIP2;2 (KJ697718), VvPnTIP4;1 (CAO44039), VvTnTIP4;1 (KJ697719). (PDF) [file pone.0102087.s001.pdf]

Figure S1. Comparison of *V. vinifera* aquaporins from Pinot noir and Touriga nacional cultivars. Alignment of deduced amino acid sequences of *Vv*TnPIP1;4 (A), *Vv*TnPIP2;1 (B), *Vv*TnPIP2;3 (C), *Vv*TnTIP1;1 (D), *Vv*TnTIP2;2 (E), *Vv*TnTIP4;1 (F) obtained from the present study (Touriga nacional) with the database variety (Pinot noir). Accession numbers of presented protein sequences are: *Vv*PnPIP1;4 (CAO39626), *Vv*TnPIP1;4 (KJ697714), *Vv*PnPIP2;1 (CAN75442), *Vv*TnPIP2;1 (KJ697715), *Vv*PnPIP2;3 (CAO18152), *Vv*TnPIP2;3 (KJ697716), *Vv*PnTIP1;1 (CAO69259), *Vv*TnTIP1;1 (KJ697717), *Vv*PnTIP2;2 (CAO23095), *Vv*TnTIP2;2 (KJ697718), *Vv*PnTIP4;1 (CAO44039), *Vv*TnTIP4;1 (KJ697719).

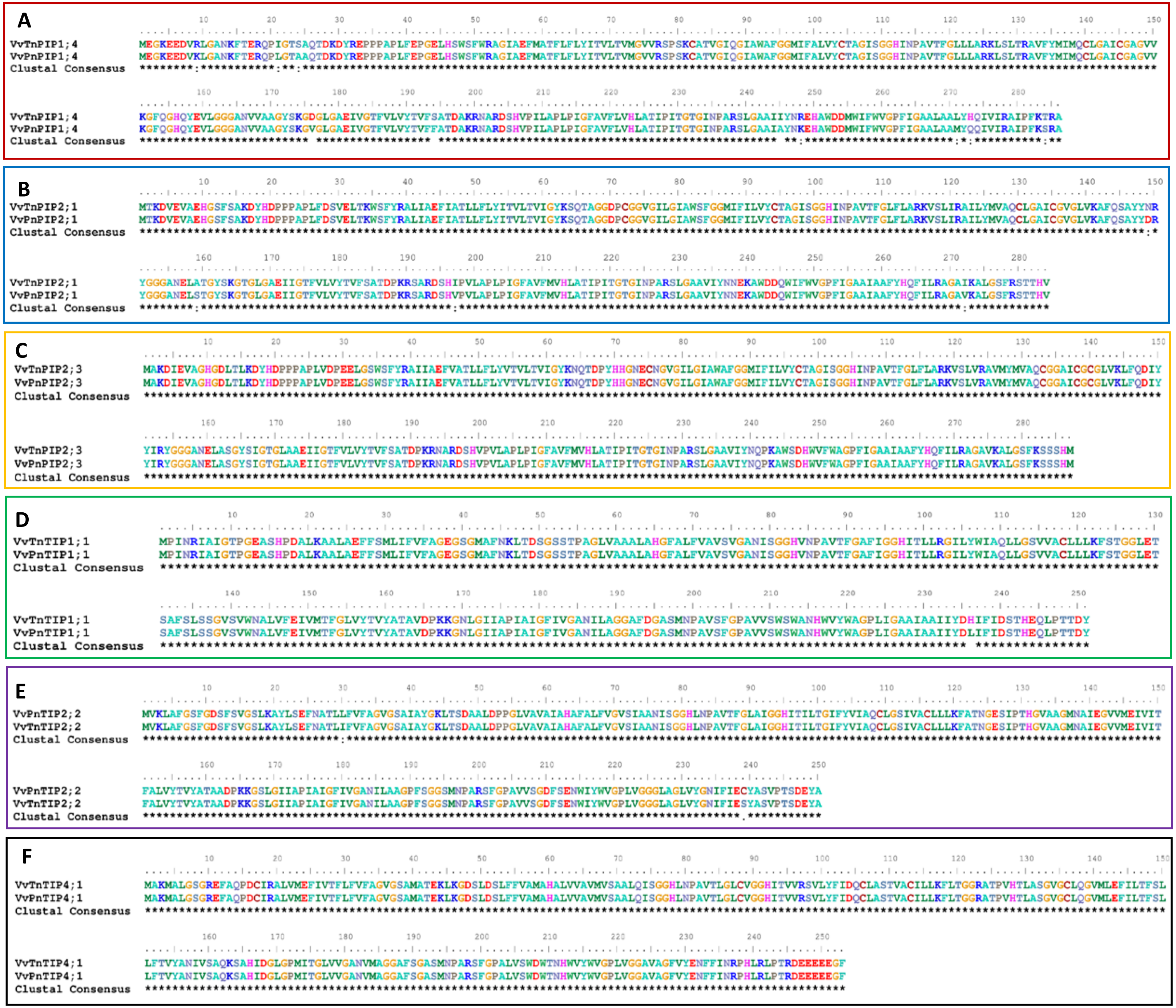
A
VvTnPIP1;4
VvPnPIP1;4
Clustal Consensus
B
VvTnPIP2;1
VvPnPIP2;1
Clustal Consensus
C
VvTnPIP2;3
VvPnPIP2;3
Clustal Consensus
D
VvTnTIP1;1
VvPnTIP1;1
Clustal Consensus
E
VvPnTIP2;2
VvTnTIP2;2
Clustal Consensus
F
VvTnTIP4;1
VvPnTIP4;1
Clustal Consensus
