# Supplementary material for: Exploring Three PIPs and Three TIPs of Grapevine for Transport of Water and Atypical Substrates through Heterologous Expression in aqy-null Yeast
Source: PLoS One. 2014 Aug 11;9(8):e102087. doi: 10.1371/journal.pone.0102087 (PMC4128642; doi:10.1371/journal.pone.0102087)

Figure S2. Comparison of N- (A) and C- (B) terminals of PIP1s, PIP2s and TIPs aquaporins. Aquaporins cloned in the present study are marked with red arrows. Accession numbers of presented protein sequences are: *At*PIP2;1 (P43286), *At*-deltaTIP2 (CAB10515), *At*-gammaTIP3 (AAC62778), *At*-epsilonTIP (AAC42249), *Fa*PIP2;1 (ADJ67992), *Pv*-alphaTIP (CAA44669), *So*PIP2;1 (4JC6_N), *Vv*PnPIP1;1 (CAO41326), *Vv*TnPIP1;1 (HQ913643), *Vv*PnPIP1;4 (CAO39626), *Vv*TnPIP1;4 (KJ697714), *Vv*PnPIP2;1 (CAN75442), *Vv*TnPIP2;1 (KJ697715), *Vv*PnPIP2;2 (CAO47394), *Vv*TnPIP2;2 (HQ913642), *Vv*PnPIP2;3 (CAO18152), *Vv*TnPIP2;3 (KJ697716), *Vv*PnTIP1;1 (CAO69259), *Vv*TnTIP1;1 (KJ697717), *Vv*PnTIP2;1 (CAO45860), *Vv*TnTIP2;1 (HQ913640), *Vv*PnTIP2;2 (CAO23095), *Vv*TnTIP2;2 (KJ697718), *Vv*PnTIP4;1 (CAO44039), *Vv*TnTIP4;1 (KJ697719), *Zm*PIP2;1 (Q84RL7), *Zm*PIP2;5 (Q9XF58). *At*: *Arabidopsis thaliana, Fa: Fragaria x ananassa, Pv*: *Phaseolus vulgaris, So*: *Spinacia oleracea, Vv*Pn: *Vitis vinifera* cv. Pinot noir, *Vv*Tn:*V. vinifera* cv. Touriga nacional.

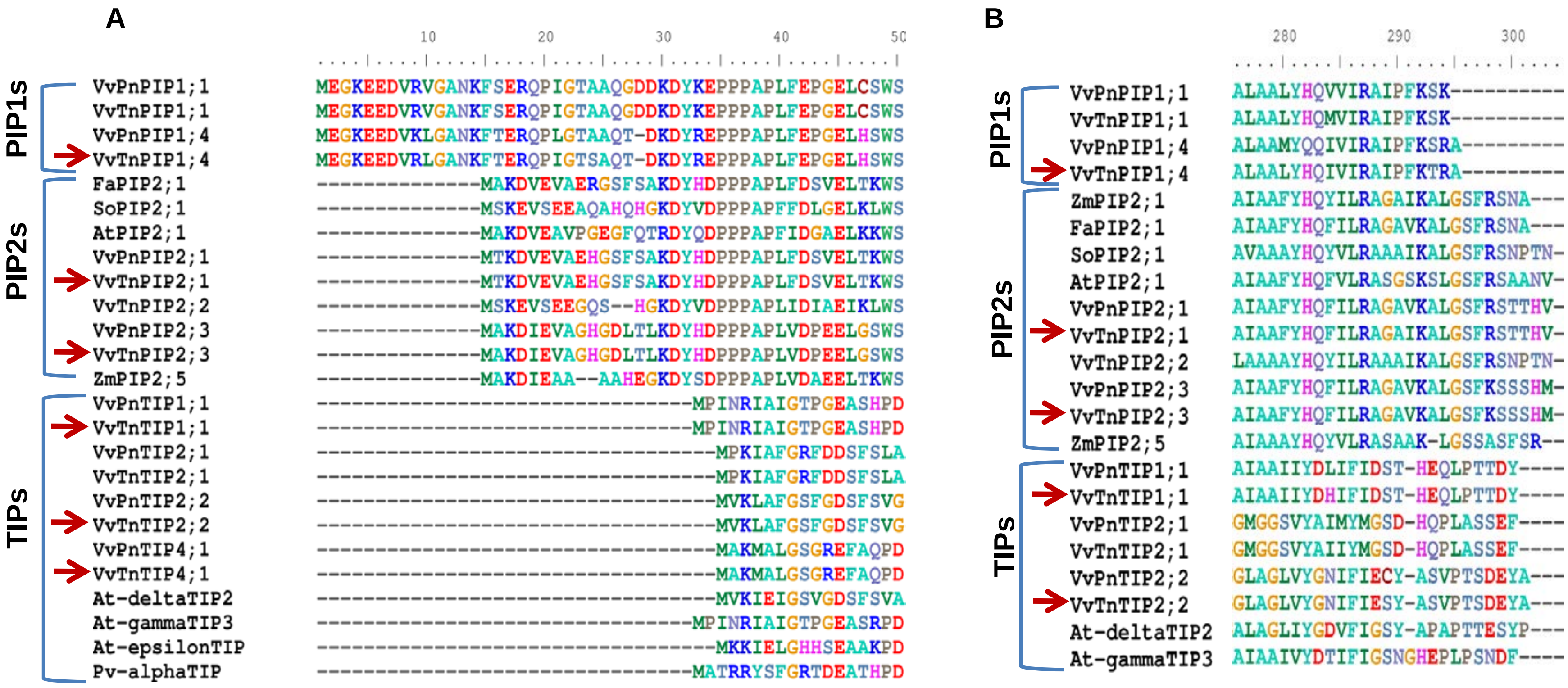

Supplement: Figure S2 — Comparison of N- (A) and C- (B) terminals of PIP1s, PIP2s and TIPs aquaporins. Aquaporins cloned in the present study are marked with red arrows. Accession numbers of presented protein sequences are: AtPIP2;1 (P43286), At-deltaTIP2 (CAB10515), At-gammaTIP3 (AAC62778), At-epsilonTIP (AAC42249), FaPIP2;1 (ADJ67992), Pv-alphaTIP (CAA44669), SoPIP2;1 (4JC6_N), VvPnPIP1;1 (CAO41326), VvTnPIP1;1 (HQ913643), VvPnPIP1;4 (CAO39626), VvTnPIP1;4 (KJ697714), VvPnPIP2;1 (CAN75442), VvTnPIP2;1 (KJ697715), VvPnPIP2;2 (CAO47394), VvTnPIP2;2 (HQ913642), VvPnPIP2;3 (CAO18152), VvTnPIP2;3 (KJ697716), VvPnTIP1;1 (CAO69259), VvTnTIP1;1 (KJ697717), VvPnTIP2;1 (CAO45860), VvTnTIP2;1 (HQ913640), VvPnTIP2;2 (CAO23095), VvTnTIP2;2 (KJ697718), VvPnTIP4;1 (CAO44039), VvTnTIP4;1 (KJ697719), ZmPIP2;1 (Q84RL7), ZmPIP2;5 (Q9XF58). At: Arabidopsis thaliana, Fa: Fragaria×ananassa, Pv: Phaseolus vulgaris, So: Spinacia oleracea, VvPn: Vitis vinifera cv. Pinot noir, VvTn: V. vinifera cv. Touriga nacional. (PDF) [file pone.0102087.s002.pdf]
