# Supplementary material for: Exploring Three PIPs and Three TIPs of Grapevine for Transport of Water and Atypical Substrates through Heterologous Expression in aqy-null Yeast
Source: PLoS One. 2014 Aug 11;9(8):e102087. doi: 10.1371/journal.pone.0102087 (PMC4128642; doi:10.1371/journal.pone.0102087)
Supplement: Figure S4 — Putative phosphorylation sites in aquaporins. Ser residue in PIPs and Thr residue in TIPs aquaporins in loop B (A), loop D (B) and C-terminal (C) are tentative phosphorylation sites. Consensus sequences are framed and conserved residues are indicated as red triangle. Aquaporins cloned in the present study are marked with red arrows. Accession numbers of presented protein sequences are: AtPIP1;2 (Q06611), AtPIP1;4 (Q39196), AtPIP2;1 (P43286), At-deltaTIP2 (CAB10515), At-gammaTIP3 (AAC62778), At-alphaTIP (AAC42249), FaPIP2;1 (ADJ67992), SoPIP2;1 (4JC6_N), VvPnPIP1;1 (CAO41326), VvTnPIP1;1 (HQ913643), VvPnPIP1;4 (CAO39626), VvTnPIP1;4 (KJ697714), VvPnPIP2;1 (CAN75442), VvTnPIP2;1 (KJ697715), VvPnPIP2;2 (CAO47394), VvTnPIP2;2 (HQ913642), VvPnPIP2;3 (CAO18152), VvTnPIP2;3 (KJ697716), VvPnTIP1;1 (CAO69259), VvTnTIP1;1 (KJ697717), VvPnTIP2;1 (CAO45860), VvTnTIP2;1 (HQ913640), VvPnTIP2;2 (CAO23095), VvTnTIP2;2 (KJ697718), VvPnTIP4;1 (CAO44039), VvTnTIP4;1 (KJ697719), ZmPIP2;1 (Q84RL7), ZmPIP2;4 (Q9ATM6), ZmPIP2;5 (Q9XF58). At: Arabidopsis thaliana, Fa: Fragaria×ananassa, Pv: Phaseolus vulgaris, So: Spinacia oleracea, VvPn: Vitis vinifera cv. Pinot noir, VvTn: V. vinifera cv. Touriga nacional, Zm: Zea mays. (PDF) [file pone.0102087.s004.pdf]

Figure S4. Putative phosphorylation sites in aquaporins. Ser residue in PIPs and Thr residue in TIPs aquaporins in loop B (A), loop D (B) and C-terminal (C) are tentative phosphorylation sites. Consensus sequences are framed and conserved residues are indicated as red triangle. Aquaporins cloned in the present study are marked with red arrows. Accession numbers of presented protein sequences are: *At*PIP1;2 (Q06611), *At*PIP1;4 (Q39196), *At*PIP2;1 (P43286), *At*-deltaTIP2 (CAB10515), *At*-gammaTIP3 (AAC62778), *At*-alphaTIP (AAC42249), *Fa*PIP2;1 (ADJ67992), *So*PIP2;1 (4JC6_N), *Vv*PnPIP1;1 (CAO41326), *Vv*TnPIP1;1 (HQ913643), *Vv*PnPIP1;4 (CAO39626), *Vv*TnPIP1;4 (KJ697714), *Vv*PnPIP2;1 (CAN75442), *Vv*TnPIP2;1 (KJ697715), *Vv*PnPIP2;2 (CAO47394), *Vv*TnPIP2;2 (HQ913642), *Vv*PnPIP2;3 (CAO18152), *Vv*TnPIP2;3 (KJ697716), *Vv*PnTIP1;1 (CAO69259), *Vv*TnTIP1;1 (KJ697717), *Vv*PnTIP2;1 (CAO45860), *Vv*TnTIP2;1 (HQ913640), *Vv*PnTIP2;2 (CAO23095), *Vv*TnTIP2;2 (KJ697718), *Vv*PnTIP4;1 (CAO44039), *Vv*TnTIP4;1 (KJ697719), *Zm*PIP2;1 (Q84RL7), *Zm*PIP2;4 (Q9ATM6), *Zm*PIP2;5 (Q9XF58). *At*: *Arabidopsis thaliana, Fa*: *Fragaria x ananassa, Pv*: *Phaseolus vulgaris*, *So*: *Spinacia oleracea, Vv*Pn: *Vitis vinifera* cv. Pinot noir, *Vv*Tn: *V. vinifera* cv. Touriga nacional, *Zm*: *Zea mays.*

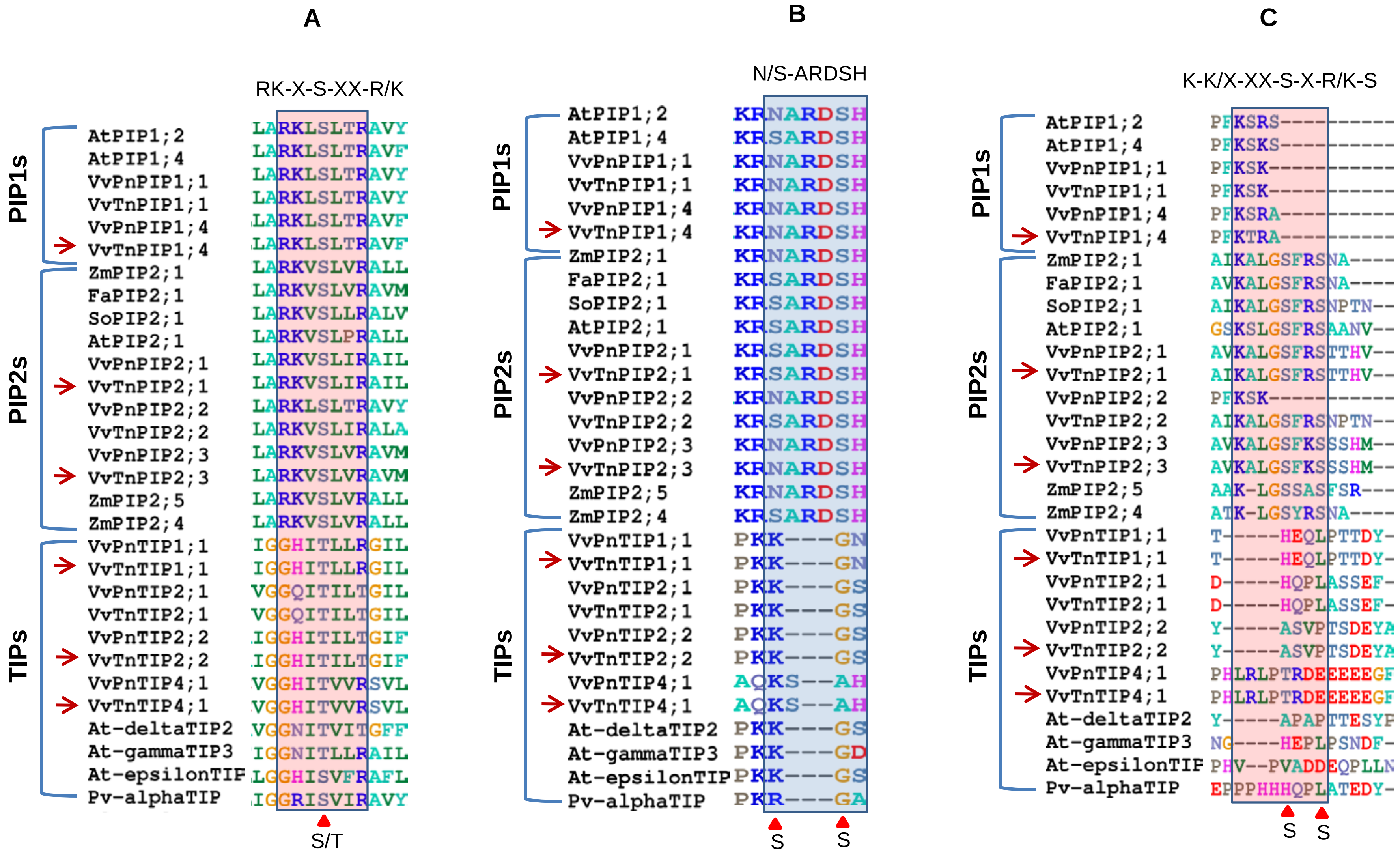
A
RK-X-S-XX-R/K
PIP1s
PIP2s
TIPs
AtPIP1;2 LARKLSLTRAVY
AtPIP1;4 LARKLSLTRAVF
VvPnPIP1;1 LARKLSLTRAVY
VvTnPIP1;1 LARKLSLTRAVY
VvPnPIP1;4 LARKLSLTRAVF
VvTnPIP1;4 LARKLSLTRAVF
ZmPIP2;1 LARKVSLVRALL
FaPIP2;1 LARKVSLVRAVM
SoPIP2;1 LARKVSLLRALV
AtPIP2;1 LARKVSLPRALL
VvPnPIP2;1 LARKVSLIRAIL
VvTnPIP2;1 LARKVSLIRAIL
VvPnPIP2;2 LARKLSLTRAVY
VvTnPIP2;2 LARKVSLIRALA
VvPnPIP2;3 LARKVSLVRAVM
VvTnPIP2;3 LARKVSLVRAVM
ZmPIP2;5 LARKVSLVRALL
ZmPIP2;4 LARKVSLVRALL
VvPnTIP1;1 IGGHITLLRGIL
VvTnTIP1;1 IGGHITLLRGIL
VvPnTIP2;1 VGGQITILTGIL
VvTnTIP2;1 VGGQITILTGIL
VvPnTIP2;2 IGGHITILTGIF
VvTnTIP2;2 IGGHITILTGIF
VvPnTIP4;1 VGGHITVVRSVL
VvTnTIP4;1 VGGHITVVRSVL
At-deltaTIP2 VGGNITVITGFF
At-gammaTIP3 IGGNITLLRAIL
At-epsilonTIP LGGHISVFRAFL
Pv-alphaTIP IGGRISVIRAVY
S/T
B
N/S-ARDSH
PIP1s
PIP2s
TIPs
AtPIP1;2 KRNARDSH
AtPIP1;4 KRSARDSH
VvPnPIP1;1 KRNARDSH
VvTnPIP1;1 KRNARDSH
VvPnPIP1;4 KRNARDSH
VvTnPIP1;4 KRNARDSH
ZmPIP2;1 KRNARDSH
FaPIP2;1 KRSARDSH
SoPIP2;1 KRSARDSH
AtPIP2;1 KRSARDSH
VvPnPIP2;1 KRSARDSH
VvTnPIP2;1 KRSARDSH
VvPnPIP2;2 KRNARDSH
VvTnPIP2;2 KRSARDSH
VvPnPIP2;3 KRNARDSH
VvTnPIP2;3 KRNARDSH
ZmPIP2;5 KRNARDSH
ZmPIP2;4 KRSARDSH
VvPnTIP1;1 PKK---GN
VvTnTIP1;1 PKK---GN
VvPnTIP2;1 PKK---GS
VvTnTIP2;1 PKK---GS
VvPnTIP2;2 PKK---GS
VvTnTIP2;2 PKK---GS
VvPnTIP4;1 AQKS--AH
VvTnTIP4;1 AQKS--AH
At-deltaTIP2 PKK---GS
At-gammaTIP3 PKK---GD
At-epsilonTIP PKK---GS
Pv-alphaTIP PKR---GA
S
S
C
K-K/X-XX-S-X-R/K-S
PIP1s
PIP2s
TIPs
AtPIP1;2 PFKSRS----------
AtPIP1;4 PFKSKS----------
VvPnPIP1;1 PFKSK-----------
VvTnPIP1;1 PFKSK-----------
VvPnPIP1;4 PFKSRA----------
VvTnPIP1;4 PFKTRA----------
ZmPIP2;1 AIKALGSFRSNA----
FaPIP2;1 AVKALGSFRSNA----
SoPIP2;1 AIKALGSFRSNPTN--
AtPIP2;1 GSKSLGSFRSAANV--
VvPnPIP2;1 AVKALGSFRSTTHV--
VvTnPIP2;1 AIKALGSFRSTTHV--
VvPnPIP2;2 PFKSK-----------
VvTnPIP2;2 AIKALGSFRSNPTN--
VvPnPIP2;3 AVKALGSFKSSSHM--
VvTnPIP2;3 AVKALGSFKSSSHM--
ZmPIP2;5 AAK-LGSSASFSR---
ZmPIP2;4 ATK-LGSYRSNA----
VvPnTIP1;1 T-----HEQLPTTDY-
VvTnTIP1;1 T-----HEQLPTTDY-
VvPnTIP2;1 D-----HQPLASSEF-
VvTnTIP2;1 D-----HQPLASSEF-
VvPnTIP2;2 Y-----ASVPTSDEYA
VvTnTIP2;2 Y-----ASVPTSDEYA
VvPnTIP4;1 PHLRLPTRDEEEEEGF
VvTnTIP4;1 PHLRLPTRDEEEEEGF
At-deltaTIP2 Y-----APAPTTESYF
At-gammaTIP3 NG----HEPLPSNDF-
At-epsilonTIP PHV--PVADDEQPLLN
Pv-alphaTIP EPPPHHHQPLATEDY-
S
S
