# Supplementary material for: Exploring Three PIPs and Three TIPs of Grapevine for Transport of Water and Atypical Substrates through Heterologous Expression in aqy-null Yeast
Source: PLoS One. 2014 Aug 11;9(8):e102087. doi: 10.1371/journal.pone.0102087 (PMC4128642; doi:10.1371/journal.pone.0102087)

Figure S5. Mercury sensitive site and pH regulation site in TIPs. Alignment of TIPs aquaporins for (A) mercury sensitive site (Cys residue) and (B) pH regulation site (His residue) for gating. Consensus sequences are framed and conserved residues are indicated as red triangle. TIPs cloned in the present study are marked with red arrows. Accession numbers of presented protein sequences are: *At*-deltaTIP2 CAB10515), *At*-gammaTIP3 (AAC62778), *At*-alphaTIP (AAC42249), *Pv*-alphaTIP (CAA44669), *Vv*PnTIP1;1 (CAO69259), *Vv*TnTIP1;1 (KJ697717), *Vv*PnTIP2;1 (CAO45860), *Vv*TnTIP2;1 (HQ913640), *Vv*PnTIP2;2 (CAO23095), *Vv*TnTIP2;2 (KJ697718), *Vv*PnTIP4;1 (CAO44039), *Vv*TnTIP4;1 (KJ697719). *At*: *Arabidopsis thaliana, Pv*: *Phaseolus vulgaris, Vv*Pn: *Vitis vinifera* cv. Pinot noir, *Vv*Tn: *V. Vinifera* (cv. Touriga nacional).

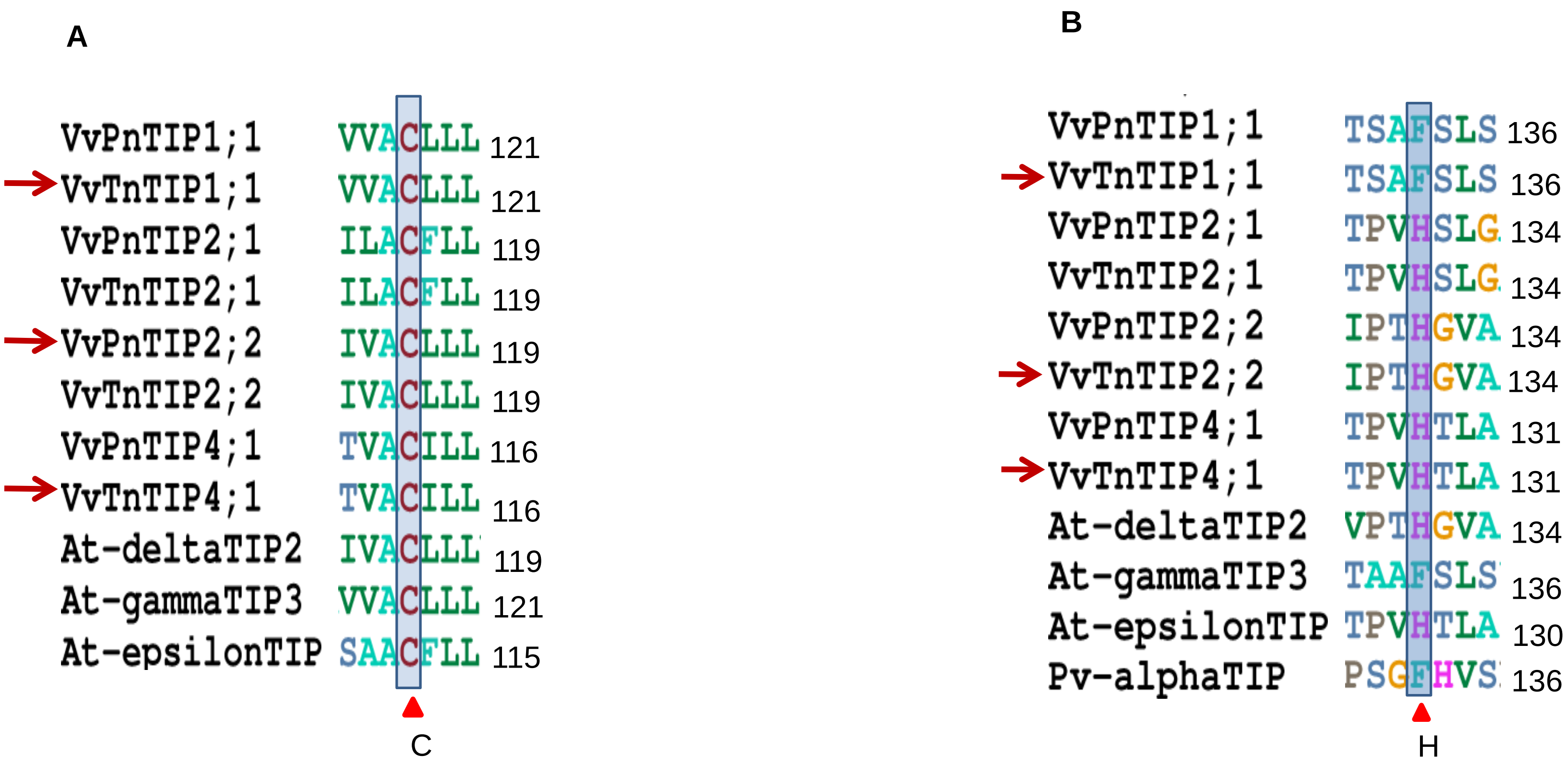

Supplement: Figure S5 — Mercury sensitive site and pH regulation site in TIPs. Alignment of TIPs aquaporins for (A) mercury sensitive site (Cys residue) and (B) pH regulation site (His residue) for gating. Consensus sequences are framed and conserved residues are indicated as red triangle. TIPs cloned in the present study are marked with red arrows. Accession numbers of presented protein sequences are: At-deltaTIP2 CAB10515), At-gammaTIP3 (AAC62778), At-alphaTIP (AAC42249), Pv-alphaTIP (CAA44669), VvPnTIP1;1 (CAO69259), VvTnTIP1;1 (KJ697717), VvPnTIP2;1 (CAO45860), VvTnTIP2;1 (HQ913640), VvPnTIP2;2 (CAO23095), VvTnTIP2;2 (KJ697718), VvPnTIP4;1 (CAO44039), VvTnTIP4;1 (KJ697719). At: Arabidopsis thaliana, Pv: Phaseolus vulgaris, VvPn: Vitis vinifera cv. Pinot noir, VvTn: V. Vinifera (cv. Touriga nacional). (PDF) [file pone.0102087.s005.pdf]
