# Supplementary material for: Exploring Three PIPs and Three TIPs of Grapevine for Transport of Water and Atypical Substrates through Heterologous Expression in aqy-null Yeast
Source: PLoS One. 2014 Aug 11;9(8):e102087. doi: 10.1371/journal.pone.0102087 (PMC4128642; doi:10.1371/journal.pone.0102087)
Supplement: Figure S6 — Consensus sequences for transport of boron and CO2. Alignment of putative amino acids of aquaporins of V. Vinifera (cv. Touriga nacional) obtained from present study and previous study [17] with the sequences of aquaporins reported to transport (A) boron and (B) CO2. ar/R constrictions and P1–P5 positions are shown to demonstrate the conserved amino acid residue. Accession numbers of presented protein sequences are: AtPIP1;2 (Q06611), AtNIP5;1 (NP_192776), HvPIP1;3 (BAA23745), HvPIP2;1 (BAA23744), NtAQP1 (O24662), OsNIP2;1 (Q6Z2T3), VvTnPIP1;1 (HQ913643), VvTnPIP1;4 (KJ697714), VvTnPIP2;1 (KJ697715), VvTnPIP2;2 (HQ913642), VvTnPIP2;3 (KJ697716), VvTnTIP1;1 (KJ697717), VvTnTIP2;1 (HQ913640), VvTnTIP2;2 (KJ697718), VvTnTIP4;1 (KJ697719), ZmPIP1;1 (Q41870). At: Arabidopsis thaliana, Hv: Hordeum vulgare, VvTn: V. vinifera (cv. Touriga nacional), Zm: Zea mays. (PDF) [file pone.0102087.s006.pdf]

Figure S6. Consensus sequences for transport of boron and CO_2. Alignment of putative amino acids of aquaporins of *V. Vinifera* (cv. Touriga nacional) obtained from present study and previous study (Leitão, 2012) with the sequences of aquaporins reported to transport (A) boron and (B) CO_2. ar/R constrictions and P1-P5 positions are shown to demonstrate the conserved amino acid residue. Accession numbers of presented protein sequences are: *At*PIP1;2 (Q06611), *At*NIP5;1 (NP_192776), *Hv*PIP1;3 (BAA23745), *Hv*PIP2;1 (BAA23744), *Nt*AQP1 (O24662), *Os*NIP2;1 (Q6Z2T3), *Vv*TnPIP1;1 (HQ913643), *Vv*TnPIP1;4 (KJ697714), *Vv*TnPIP2;1 (KJ697715), *Vv*TnPIP2;2 (HQ913642), *Vv*TnPIP2;3 (KJ697716), *Vv*TnTIP1;1 (KJ697717), *Vv*TnTIP2;1 (HQ913640), *Vv*TnTIP2;2 (KJ697718), *Vv*TnTIP4;1 (KJ697719), *Zm*PIP1;1 (Q41870). *At*: *Arabidopsis thaliana, Hv: Hordeum vulgare, Vv*Tn: *V. vinifera* (cv. Touriga nacional)*, Zm*: *Zea mays.*

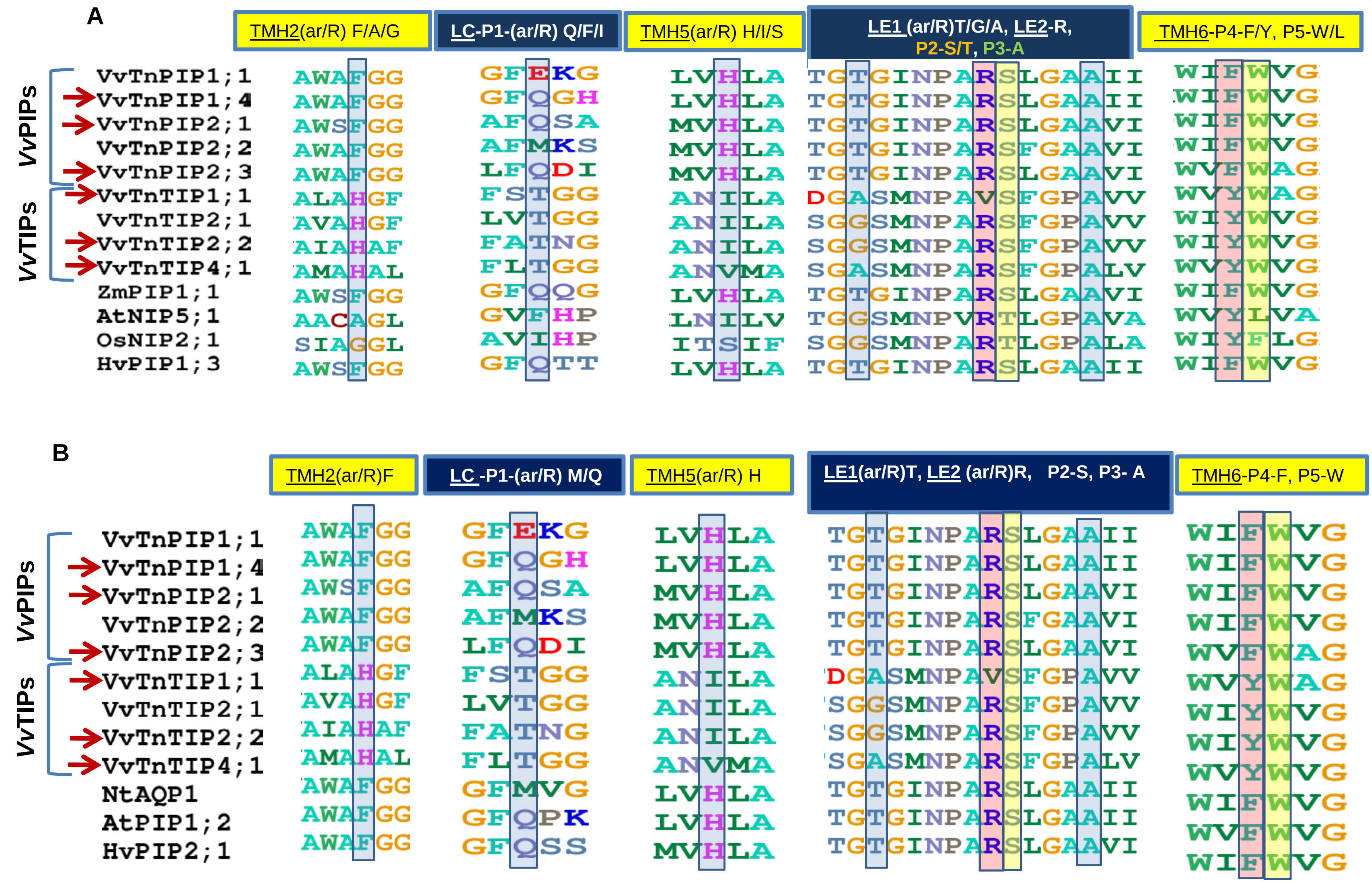

Supplementary references

Leitão L, Prista C, Moura TF, Loureiro-Dias MC, Soveral G (2012) Grapevine aquaporins: gating of a tonoplast intrinsic protein (TIP2;1) by cytosolic pH. PLoS One 7: e33219.
