# Supplementary material for: Exploring Three PIPs and Three TIPs of Grapevine for Transport of Water and Atypical Substrates through Heterologous Expression in aqy-null Yeast
Source: PLoS One. 2014 Aug 11;9(8):e102087. doi: 10.1371/journal.pone.0102087 (PMC4128642; doi:10.1371/journal.pone.0102087)
Supplement: Figure S7 — Consensus sequences for transport of ammonia. Alignment of putative amino acids of aquaporins of V. Vinifera (cv. Touriga nacional) obtained from present study and previous study [17] with sequences of aquaporins reported to transport ammonia. ar/R constrictions and P1–P5 positions are shown to demonstrate the conserved amino acid residue. Accession numbers of presented protein sequences are: AtTIP2;1 (Q41951), AtTIP2;3 (Q9FGL2), GmNOD26 (P08995), TaTIP2;1 (AAS19468),VvTnPIP1;1 (HQ913643), VvTnPIP1;4 (KJ697714), VvTnPIP2;1 (KJ697715), VvTnPIP2;2 (HQ913642), VvTnPIP2;3 (KJ697716), VvTnTIP1;1 (KJ697717), VvTnTIP2;1 (HQ913640), VvTnTIP2;2 (KJ697718), VvTnTIP4;1 (KJ697719). At: Arabidopsis thaliana, Gm: Glycine max, Ta: Triticum aestivum, VvTn: V. Vinifera (cv. Touriga nacional). (PDF) [file pone.0102087.s007.pdf]

Figure S7. Consensus sequences for transport of ammonia. Alignment of putative amino acids of aquaporins of *V. Vinifera* (cv. Touriga nacional) obtained from present study and previous study (Leitão, 2012) with sequences of aquaporins reported to transport ammonia. ar/R constrictions and P1-P5 positions are shown to demonstrate the conserved amino acid residue. Accession numbers of presented protein sequences are: *At*TIP2;1 (Q41951), *At*TIP2;3 (Q9FGL2), *Gm*NOD26 (P08995), *Ta*TIP2;1 (AAS19468),*Vv*TnPIP1;1 (HQ913643), *Vv*TnPIP1;4 (KJ697714), *Vv*TnPIP2;1 (KJ697715), *Vv*TnPIP2;2 (HQ913642), *Vv*TnPIP2;3 (KJ697716), *Vv*TnTIP1;1 (KJ697717), *Vv*TnTIP2;1 (HQ913640), *Vv*TnTIP2;2 (KJ697718), *Vv*TnTIP4;1 (KJ697719). *At*: *Arabidopsis thaliana, Gm*: *Glycine max*, *Ta*: *Triticum aestivum, Vv*Tn: *V. Vinifera* (cv. Touriga nacional).

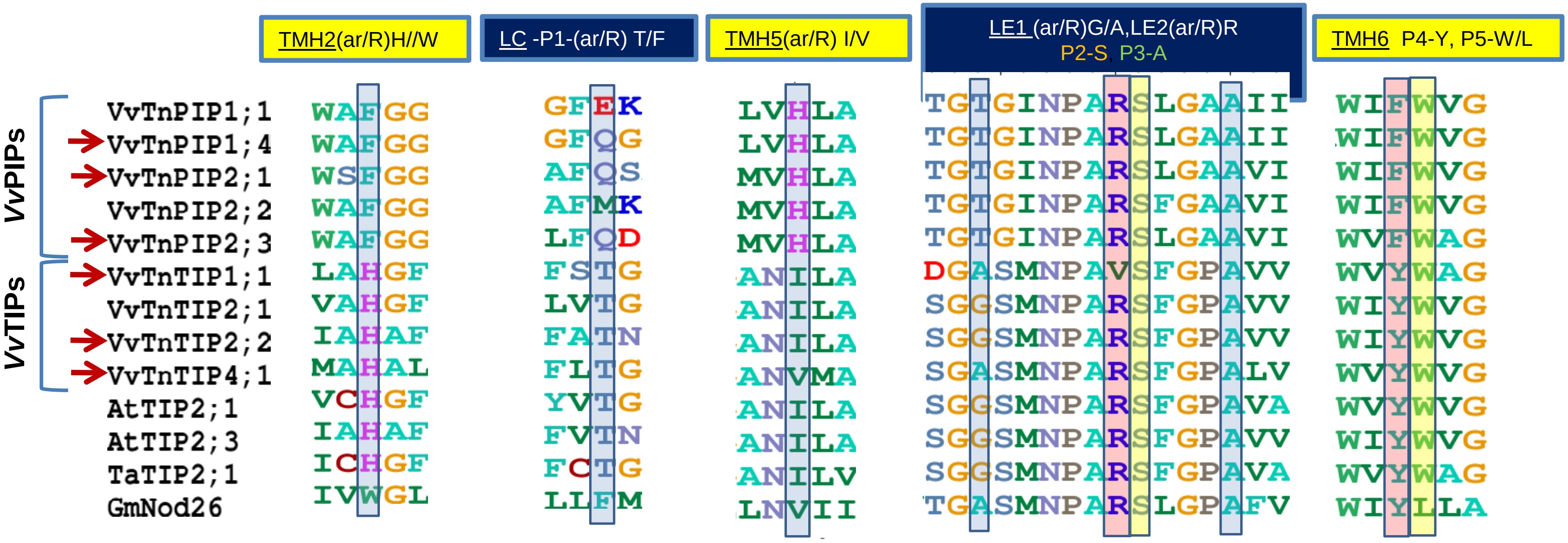

Supplementary references

Leitão L, Prista C, Moura TF, Loureiro-Dias MC, Soveral G (2012) Grapevine aquaporins: gating of a tonoplast intrinsic protein (TIP2;1) by cytosolic pH. PLoS One 7: e33219.
