# Supplementary material for: Exploring Three PIPs and Three TIPs of Grapevine for Transport of Water and Atypical Substrates through Heterologous Expression in aqy-null Yeast
Source: PLoS One. 2014 Aug 11;9(8):e102087. doi: 10.1371/journal.pone.0102087 (PMC4128642; doi:10.1371/journal.pone.0102087)
Supplement: Figure S8 — Consensus sequences for transport of H2O2 and urea. Alignment of putative amino acids of aquaporins of V. Vinifera (cv. Touriga nacional) obtained from present study and previous study [17] with sequences of aquaporins reported to transport (A) H2O2 and (B) urea. ar/R constrictions and P1–P5 positions are shown to demonstrate the conserved amino acid residue. Accession numbers of presented protein sequences are: AtTIP1;1 (P25818), AtTIP1;2 (Q41963), AtTIP1;3 (NP_192056), AtTIP2;1 (Q41951), AtTIP2;3 (Q9FGL2), AtPIP2;4 (Q9FF53), CpNIP1 (CAD67694), NtAQP1 (O24662), NtTIPa (Q9XG70), OsNIP2;1 (Q6Z2T3), VvTnPIP1;1 (HQ913643), VvTnPIP1;4 (KJ697714), VvTnPIP2;1 (KJ697715), VvTnPIP2;2 (HQ913642), VvTnPIP2;3 (KJ697716), VvTnTIP1;1 (KJ697717), VvTnTIP2;1 (HQ913640), VvTnTIP2;2 (KJ697718), VvTnTIP4;1 (KJ697719), ZmPIP1;5 (Q9AR14). At: Arabidopsis thaliana, Cp: Cucurbita pepo, Nt: Nicotiana tabacum, Os: Oryza sativa, VvTn: Vitis vinifera (cv. Touriga nacional), Zm: Zea mays. (PDF) [file pone.0102087.s008.pdf]

Figure S8. Consensus sequences for transport of H_2O_2 and urea. Alignment of putative amino acids of aquaporins of *V. Vinifera* (cv. Touriga nacional) obtained from present study and previous study (Leitão, 2012) with sequences of aquaporins reported to transport (A) H_2O_2 and (B) urea. ar/R constrictions and P1-P5 positions are shown to demonstrate the conserved amino acid residue. Accession numbers of presented protein sequences are: *At*TIP1;1 (P25818), *At*TIP1;2 (Q41963), *At*TIP1;3 (NP_192056), *At*TIP2;1 (Q41951), *At*TIP2;3 (Q9FGL2), *At*PIP2;4 (Q9FF53), *Cp*NIP1 (CAD67694), *Nt*AQP1 (O24662), *Nt*TIPa (Q9XG70), *Os*NIP2;1 (Q6Z2T3), *Vv*TnPIP1;1 (HQ913643), *Vv*TnPIP1;4 (KJ697714), *Vv*TnPIP2;1 (KJ697715), *Vv*TnPIP2;2 (HQ913642), *Vv*TnPIP2;3 (KJ697716), *Vv*TnTIP1;1 (KJ697717), *Vv*TnTIP2;1 (HQ913640), *Vv*TnTIP2;2 (KJ697718), *Vv*TnTIP4;1 (KJ697719), *Zm*PIP1;5 (Q9AR14). *At*: *Arabidopsis thaliana, Cp: Cucurbita pepo*, *Nt*: *Nicotiana tabacum, Os*: *Oryza sativa, Vv*Tn*: Vitis vinifera* (cv. Touriga nacional)*, Zm*: *Zea mays.*

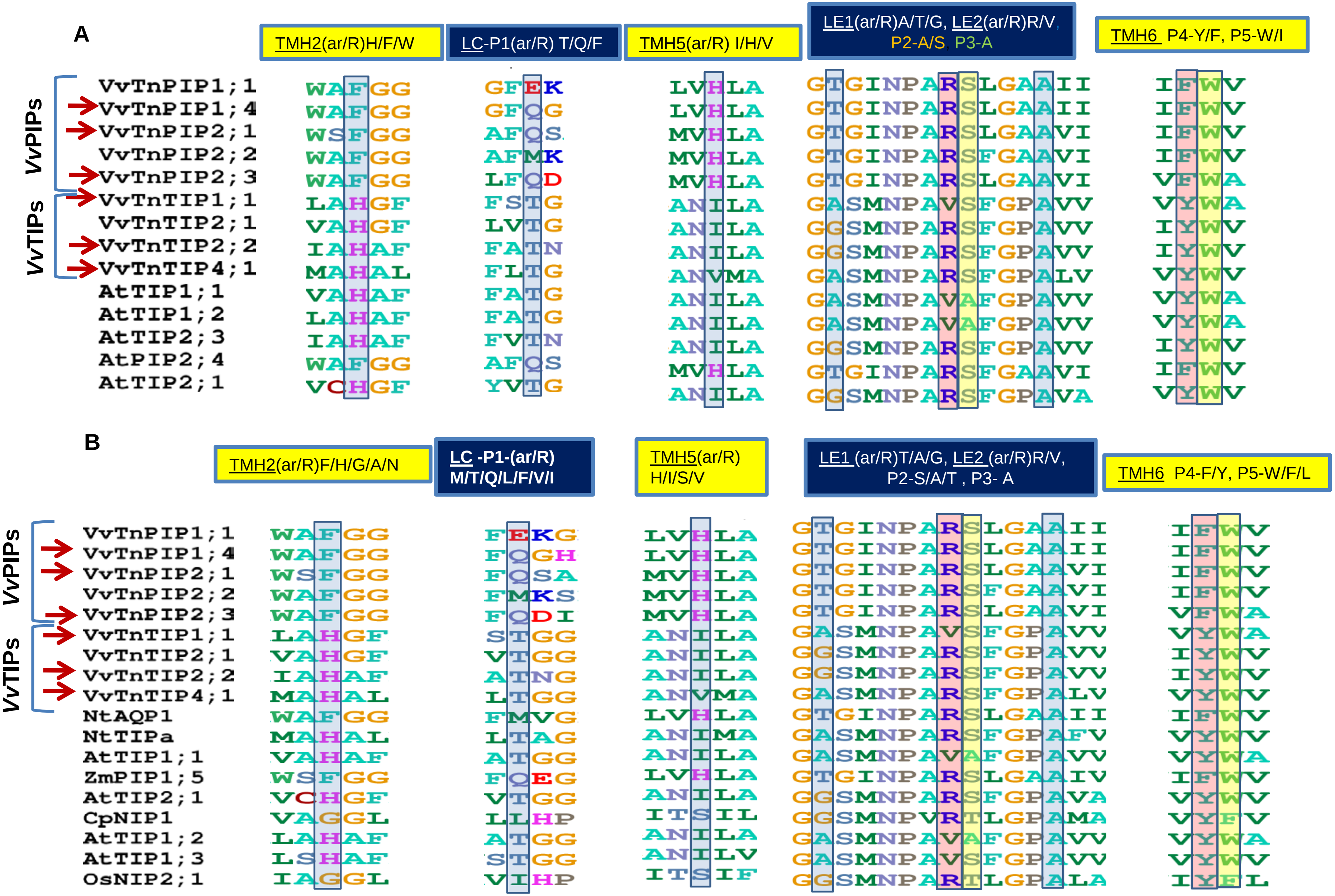

Supplementary references

Leitão L, Prista C, Moura TF, Loureiro-Dias MC, Soveral G (2012) Grapevine aquaporins: gating of a tonoplast intrinsic protein (TIP2;1) by cytosolic pH. PLoS One 7: e33219.
