# Supplementary material for: Exploring Three PIPs and Three TIPs of Grapevine for Transport of Water and Atypical Substrates through Heterologous Expression in aqy-null Yeast
Source: PLoS One. 2014 Aug 11;9(8):e102087. doi: 10.1371/journal.pone.0102087 (PMC4128642; doi:10.1371/journal.pone.0102087)
Supplement: Table S1 — Primers used in this work (restriction sites are underlined). (PDF) [file pone.0102087.s009.pdf]

Table S1. Primers used in this work (restriction sites are underlined).

Genes to amplify	Primer name	Primer sequence (5´-3´)	Restriction enzyme
PIP1;4	P14 Frwd	GCGAGCTCTAGACTCAGAAAAATGGAGGGGAAGGAAGAGGATGTGAGG	*Xba* I
	P14 Rev	ATACGCGTCGACGGCTCTGGTCTTGAATGGGATAGC	*Sal* I
PIP2;1	P21 Frwd	GGTAGCTCTAGAATGACTAAGGACGTTGAGGTTGCG	*Xba* I
	P21 Rev	ATACGCGTCGACTACATGAGTGGTGCTCCTG	*Sal* I
PIP2;3	P23 Frwd	AGTAGCTCTAGAATGGCCAAGGACATTGAGGTTGCAGG	*Xba* I
	P23 Rev	TACACCATCGATCATGTGGGAACTGCTCTTGAAAGACCCTAGAG	*Cla* I
TIP1;1	T11 Frwd	GCGAGCTCTAGAAGGGCAAGCCTTTAATAAAAATGCC	*Xba* I
	T11 Rev	GTACGCGTCGACATAATCCGTGGTGGGCAGTT	*Sal* I
TIP2;2	T22 Frwd	GCGAGCTCTAGATGACCAAAAATGGTGAAGCTTGCCTTTGGTAGCTTTGG	*Xba* I
	T22 Rev	GTACGCGTCGACAGCATATTCATCGGAGGTGG	*Sal* I
TIP4;1	T41 Frwd	GCGAGCTCTAGACATTCCTCTCTCAACATGGCCAAGATG	*Xba* I
	T41 Rev	GTACGCGTCGACGAAGCCTTCTTCTTCTTCTTCGTCTC	*Sal* I
