# Supplementary material for: Exploring Three PIPs and Three TIPs of Grapevine for Transport of Water and Atypical Substrates through Heterologous Expression in aqy-null Yeast
Source: PLoS One. 2014 Aug 11;9(8):e102087. doi: 10.1371/journal.pone.0102087 (PMC4128642; doi:10.1371/journal.pone.0102087)

Figure S3. Presence of methylation and sorting signal sequences (D/E-X-D/E) at N-terminal (A) and pH regulation site (His residue) for gating (B) of PIPs aquaporins. Consensus sequences are framed and conserved residues are indicated as red triangle. Aquaporins cloned in the present study are marked with red arrows. Accession numbers of presented protein sequences are: *At*PIP2;1 (P43286), *At*PIP1;2 (Q06611), *At*PIP1;4 (Q39196), *Fa*PIP2;1 (ADJ67992), *So*PIP2;1 (4JC6_N), *Vv*PnPIP1;1 (CAO41326), *Vv*TnPIP1;1 (HQ913643), *Vv*PnPIP1;4 (CAO39626), *Vv*TnPIP1;4 (KJ697714), *Vv*PnPIP2;1 (CAN75442), *Vv*TnPIP2;1 (KJ697715), *Vv*PnPIP2;2 (CAO47394), *Vv*TnPIP2;2 (HQ913642), *Vv*PnPIP2;3 (CAO18152), *Vv*TnPIP2;3 (KJ697716), *Zm*PIP2;1 (Q84RL7), *Zm*PIP2;5 (Q9XF58), *Zm*PIP2;4 (Q9ATM6). *At*: *Arabidopsis thaliana, Fa*: *Fragaria x ananassa,* So: *Spinacia oleracea, Vv*Pn: *Vitis vinifera* cv. Pinot noir, *Vv*Tn: *V. vinifera* cv. Touriga nacional, *Zm*: *Zea mays.*

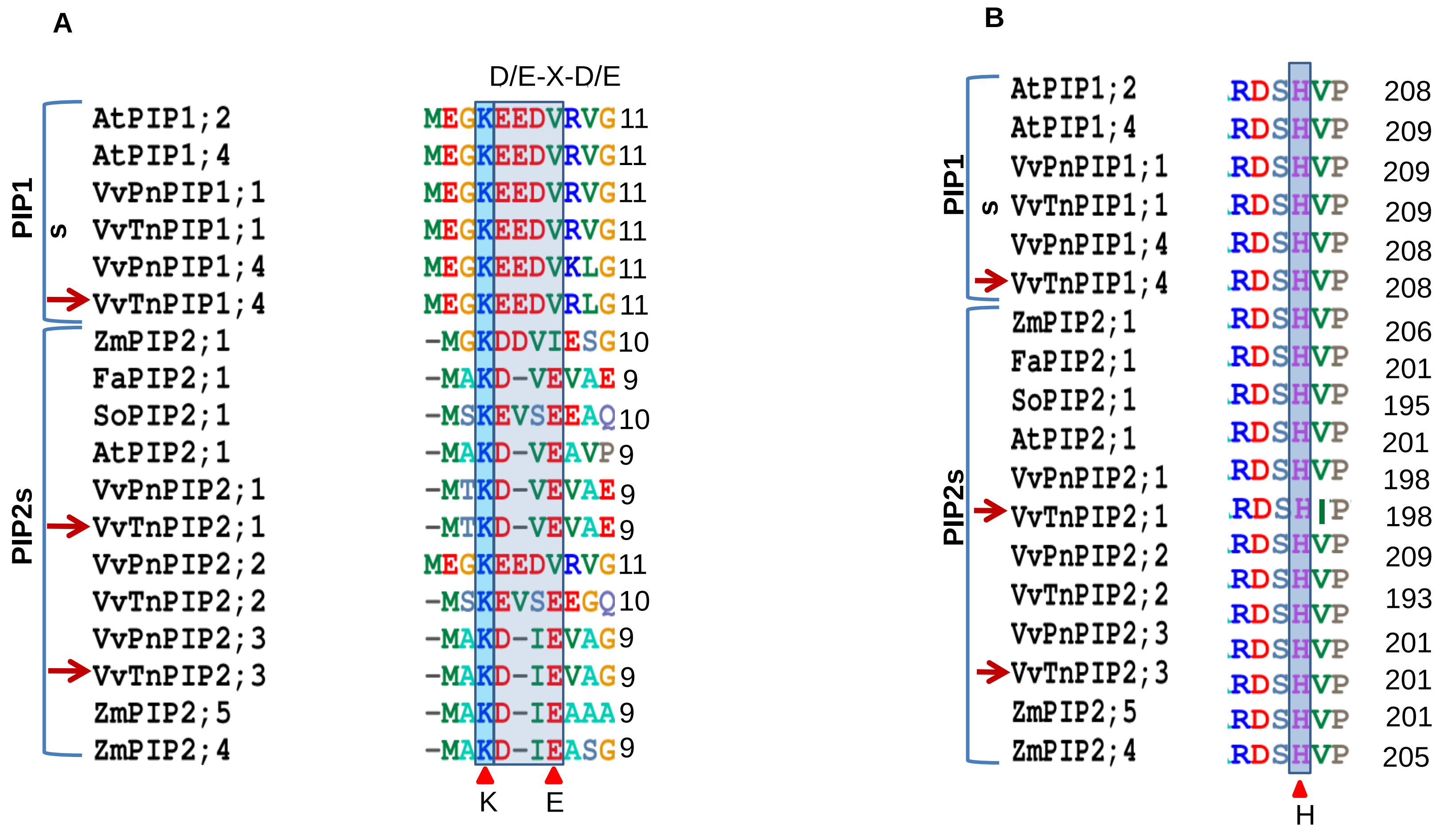

Supplement: Figure S3 — Presence of methylation and sorting signal sequences (D/E-X-D/E) at N-terminal (A) and pH regulation site (His residue) for gating (B) of PIPs aquaporins. Consensus sequences are framed and conserved residues are indicated as red triangle. Aquaporins cloned in the present study are marked with red arrows. Accession numbers of presented protein sequences are: AtPIP2;1 (P43286), AtPIP1;2 (Q06611), AtPIP1;4 (Q39196), FaPIP2;1 (ADJ67992), SoPIP2;1 (4JC6_N), VvPnPIP1;1 (CAO41326), VvTnPIP1;1 (HQ913643), VvPnPIP1;4 (CAO39626), VvTnPIP1;4 (KJ697714), VvPnPIP2;1 (CAN75442), VvTnPIP2;1 (KJ697715), VvPnPIP2;2 (CAO47394), VvTnPIP2;2 (HQ913642), VvPnPIP2;3 (CAO18152), VvTnPIP2;3 (KJ697716), ZmPIP2;1 (Q84RL7), ZmPIP2;5 (Q9XF58), ZmPIP2;4 (Q9ATM6). At: Arabidopsis thaliana, Fa: Fragaria×ananassa, So: Spinacia oleracea, VvPn: Vitis vinifera cv. Pinot noir, VvTn: V. vinifera cv. Touriga nacional, Zm: Zea mays. (PDF) [file pone.0102087.s003.pdf]
